# Accurate Prediction of Prognosis by Integrating Clinical and Molecular Characteristics in Colon Cancer

**DOI:** 10.3389/fcell.2021.664415

**Published:** 2021-05-21

**Authors:** Liru Wang, Mu Su, Mengyan Zhang, Hongyan Zhao, Hongli Wang, Jie Xing, Chenyu Guo, Dianshuang Zhou, Wenhui Xue, Haibo Lu, Yan Zhang

**Affiliations:** ^1^School of Life Science and Technology, Computational Biology Research Center, Harbin Institute of Technology, Harbin, China; ^2^Department of Medical Oncology, Heilongjiang Provincial Hospital, Harbin, China; ^3^Department of Gastroenterology, The Fourth Affiliated Hospital of Harbin Medical University, Harbin, China; ^4^Department of Gastrointestinal Medical Oncology, Harbin Medical University Cancer Hospital, Harbin, Heilongjiang, China; ^5^Guangzhou Institute of Respiratory Health, State Key Laboratory of Respiratory Disease, Guangzhou Medical University, Guangzhou, China

**Keywords:** prognostic model, combination, conlon cancer, clinical feature, molecular characteristic

## Abstract

Various factors affect the prognosis of patients with colon cancer. Complicated factors are found to be conducive to accurate assessment of prognosis. In this study, we developed a series of prognostic prediction models for survival time of colon cancer patients after surgery. Analysis of nine clinical characteristics showed that the most important factor was the positive lymph node ratio (LNR). High LNR was the most important clinical factor affecting 1- and 3-year survival; M0&age < 70 was the most important feature for 5 years. The performance of the model was improved through the integration of clinical characteristics and four types of molecule features (mRNA, lncRNA, miRNA, DNA methylation). The model provides guidance for clinical practice. According to the high-risk molecular features combined with age ≥ 70&T3, poorly differentiated or undifferentiated, M0&well differentiated, M0&T2, LNR high, T4&poorly differentiated, or undifferentiated, the survival time may be less than 1 year; for patients with high risk of molecular features combined with M0&T2, M0&T4, LNR 0& M0, LNR median &T3, and LNR high, the survival is predicted less than 3 years; and the survival of patients with M1&T3, M0 and high risk molecular features is less than 5 years. Using multidimensional and complex patient information, this study establishes potential criteria for clinicians to evaluate the survival of patients for colon cancer.

## Introduction

Colon cancer is one of the most common malignancies worldwide. According to the CONCORD project’s latest survey of colon cancer in 65 countries around the world, the survival rate of colon cancer patients is approximately 50–70%, and in a small number of countries it is less than 50% for 5 years (Allemani et al., 2018). Early detection (Labianca et al., 2013), timely surgical resection, effective chemotherapy (Andre et al., 2015), and targeted therapy (Weeks et al., 1998; Robin et al., 2011) have prolonged the survival time of colon cancer patients to a certain extent (Brenner et al., 2014), but these strategies are costly. Therefore, early diagnosis and the identification of prognosis and predictive biomarkers are critically required.

Clinical features such as tumor stage and pathology have been used to guide the treatment and prognosis of colon cancer. However, on account of individual differences, establishing a standard criterion for prognostic evaluation has been difficult. With the advancement of molecular biology techniques, several prognostic-related tumor biomarkers in colon cancer have been found. Most studies on biomarkers for colon cancer have focused on somatic mutations (Eklof et al., 2013), but with the development of high-throughput sequencing, some new tumor markers have been discovered, including lncRNAs (Saus et al., 2016; Kita et al., 2017), mRNAs (Dalerba et al., 2016), miRNAs (Zhang et al., 2013; Perez-Carbonell et al., 2015), and DNA methylation events (Weisenberger et al., 2006). Most studies on the correlations are limited to a single molecular level, but many molecular changes are closely related to clinical features. For example, studies have found that miRNA biomarkers are associated with T1 colon cancer metastasis to lymph nodes (Ozawa et al., 2018). Therefore, a genome-wide analysis with a larger sample is required to construct a prognostic model to provide clinicians with tools to accurately predict the prognosis of colon cancer.

This study is based on machine learning and statistical methods to construct the prognosis model by the clinical characteristics from a large sample of the Surveillance, Epidemiology and End Results database (SEER)^1^ and the clinical/molecular features from the Cancer Genome Atlas database (TCGA)^2^ (Cancer Genome Atlas Research Network et al., 2013). After construction and validation, the results showed different survival times between high- and low-risk groups by combining clinical factors and molecular features. This study can help clinicians make decisions and improve the prognosis of colon cancer patients.

## Materials and Methods

### Data Source

Patients diagnosed with adenocarcinoma who underwent surgery for colon cancer were selected from the SEER database from April 2000 to April 2013. Data on the following nine clinical characteristics were collected: Sex, age, degree of differentiation, number of lymph nodes, number of positive lymph nodes, tumor location, primary tumor (T), regional lymph nodes (N), and distant metastasis (M). We deleted the uncertain data such as T0, Tis, Tx, N3, Nx, Nxa, Nxr, and Mx. A total of 161,694 patients were finally selected. The training samples, test samples and additional test samples of colon cancer were randomly divided into three groups at a ratio of 70, 15, and 15%.

Molecular omics datasets were obtained from the TCGA database using UCSC Xena^3^, including RNA profiles quantified as fragments per kilobase of exon per million reads mapped (FPKM) (469 cancers and 41 normal), miRNA profiles quantified as Reads of exon model per Million mapped reads (RPM) (459 cancers and eight normal), and DNA methylation profiles generated by the Illumina Infinium Human Methylation 450 Bead Chip (307 cancers and 38 normal). lncRNA and mRNA were united as log2(fpkm+1) and miRNA was united as log2(RPM+1). The samples were randomly divided into training samples and test samples at a ratio of 70 and 30%.

### Determination of Positive Lymph Node Ratio (LNR)

The positive LNR refers to the ratio of positive lymph nodes to the total number of lymph nodes. The Gain method was used to determine the positive LNR; the values range from 0 to 1 by incremental steps of 0.1 and are repeated 100,000 times. The positive LNR threshold was defined as the mean value, according to all values which were divided to four groups as 0, low, median, and high.

The method can be described in detail through the following six steps. Step1: Divide training samples and test samples according to the ratio of 2:1. Step2: Remove samples with 0 positive lymph nodes. Step3: From 0 to 1 in the set with a step size of 0.01, traverse and pick two values as the threshold of positive lymph node ratio to obtain the information gain. Step4: Obtain the maximum information gain of the threshold for positive lymph node ratio. Step5: Go back to Step1 until repeated 100,000 times. Step6: Obtain the average values of the best positive lymph node ratio threshold which are the final threshold values of the positive lymph node ratio. Therefore, the final thresholds were 0 and the other two thresholds which were gained by information gain method for the ratio of positive lymph nodes. Based on these three values, the samples were divided into four groups which were 0, low, median, and high.

### Screening Clinical Characteristics

The positive LNR threshold was determined, and univariable and multivariable Cox regression models were used to analyze the relationship between clinical characteristics and survival time. The important rankings of clinical features were, respectively, obtained using Naive Bayes, Generalized linear model, Linear discriminate model, Glmnet, and Quadratic discriminate model by R packages ‘‘caret’’(version 4.0.1)^4^ (M Kuhn et al., 2016). The average values of the importance rankings of the five classifiers for these clinical features were selected as the final importance rankings of the clinical features. The top five clinical features were regarded as the most important clinical features. Although these five classifiers are based on the idea of probability or linear regression, they still have some differences. The Naive Bayes classifier is a conditional probability model based on Bayes’ theory. The generalized linear model is a more flexible linear model, and it has not very strict distribution requirements allowing error distribution. The linear discriminant model is linear discriminant analysis which finds a linear combination of the features for two objects. The glmnet of R package “caret” is a binomial logistic regression model, and it uses a logistic function to predict a binary variable. Quadratic discriminant analysis is similar to linear discriminant analysis, but it can form a non-linear boundary by Gaussian distribution. We hope that we can avoid some overfitting problems by using a variety of similar but different classifiers. Lasso Cox regression analysis (Gao et al., 2010) needed to be repeated 1,000 times; the clinical combinations with a higher frequency than average were selected. In this study, four machine learning methods were used as prognostic prediction models for the training set, test set and additional test set, and the final number of the clinical combinations was small and the best.

### Screening Molecular Characteristics

lncRNA and mRNA were united as log2(fpkm+1), and miRNA was united as log2(RPM+1). Before gaining differentially expressed genes, we did some data preprocessing. For mRNA, miRNA, and lncRNA, the 0 values which were more than 70% of genes were removed and the remaining 0 values were replaced by the minimum value of the data set. For DNA methylation sites, the missing values which were more than 70% of genes were removed and missing values of remaining genes were recalculated. The function knnImputation, R package “DMwR,” and R function scale() were applied for normalization and standardization, when we integrated different types of molecules.

Univariable and multivariable Cox regression models were used to analyze the relationship between molecular features and survival time. The Boruta method (Shi et al., 2019) was used to select more important features for the mRNA, lncRNA, and DNA methylation sites.

Differentially expressed genes (mRNAs, miRNAs, lncRNAs) and differential DNA methylation sites between sets (cancer samples and paracancerous control samples) were identified using a two-sided *t*-test and the Benjamini–Hochberg method, which were performed to adjusted *p*-values by multiple tests. Significant differentially expressed mRNAs, miRNAs, and lncRNAs were defined when the *P*-value was less than 0.05 and fold change was greater than 2 or less than 1/2. Significant differential DNA methylation sites were defined when the adjusted *p-*value was less than 0.05 and the △β value was greater than 20 percentage points between sample pairs.

### Relationship Between Molecular Features and Clinical Features

To explore the correlation between prognosis-related molecules and clinical features, each of the obtained prognosis-related molecules was integrated into clinical features independently, and the division effect was evaluated by the ROC curve area of the Generalized Linear Model, Linear Discriminant Model, Naive Bayes Model, Glmnet, and Quadratic Discriminate Model. We also explored whether each clinical feature showed a significant difference between the high- and low-risk groups. The log-rank test was used to compare differences in the survival curve. These were implemented using R packages “caret”, “survival”, and “survcomp”.

### Model Evaluation Index

A variety of indicators were applied to test the strengths and weaknesses of the model. The R package pROC (Robin et al., 2011) was used to obtain the ROC curve area, the R package “survivalROC” (Heagerty and Zheng, 2005) was used for independent time ROC curve analysis, and concordance index (c-index) and the nomograms consisting of independent prognostic factors were also constructed based on multivariable progressive Cox regression results by employing “rms” R package.

### Statistical Analysis

Survival analyses were performed by *Kaplan--Meier* survival plot. All risk scores were calculated by a step multivariable Cox regression model, and low-risk and high-risk groups were divided according to the median risk score. Statistical analysis was performed using R statistical software version 3.5 (version x64 3.5.1)^5^.

## Results

### Constructing Colon Cancer Prognostic Prediction Models Based on Combinations of Clinical Characteristics

A total of 161,694 patients with complete data for nine clinical characteristics were obtained from SEER. Using the information gain method, cutoffs for the four groups are as listed in Methods (see comment in the above section); the positive LNR was defined as 0, 0.2, and 0.6 for 1 year or 3 years, and 0, 0.3, and 0.7 for 5 years, as described in section “Materials and Methods.” Patients divided into four groups according to LNR thresholds showed different survival outcomes by Kaplan–Meier survival curve analysis. Patients with a higher LNR showed poor survival (Supplementary Figures 1A–C, 2A–C, 3A–C). We evaluated survival and death rates according to the nine clinical characteristics. LNR high, M1, and N2 were three strong indicators of increased mortality at 1 and 3 years (Supplementary Figures 1D, 2D). LNR high, M1, and tumor location (left half of the intestine) were the top three characteristics of mortality at 5 years (Supplementary Figure 3D).

Using univariable and multivariable Cox regression analyses, we found that clinical characteristics including tumor location, sex, and N impacted the 1-year survival time. For the 3-year survival rate, sex, N, and number of acquired lymph nodes were not significant, while all clinical characteristics were markedly for the 5-year survival rate (Supplementary Figures 1–3E and Table 1). The top five important clinical features were obtained as described in section “Materials and Methods”; LNR, age, M, T, and tumor differentiation were the top five for 1-year and 3-year survival (Supplementary Figures 1F, 2F), while positive LNR, age, M, T, and N were the top five for 5-year survival (Supplementary Figure 3F). After permutation and combination of the five important clinical features, 899 feature sets were obtained and 22, 20, and 18 features were acquired by the dimension reduction of the classification model in 1, 3, or 5 years, respectively. In the training set, test set, and additional test set, the maximum AUC values of the 1-year model were 0.743, 0.748, and 0.747, respectively (Supplementary Figure 1G). The AUC values of the 3-year model were 0.718, 0.718, and 0.719, respectively (Supplementary Figure S2G). In the 5-year model, the AUC values were 0.7, 0.704, and 0.701, respectively (Supplementary Figure 3G). Age < 70 and M0 were the most significant factors with 1-, 3-, and 5-year survival.

**TABLE 1:** Multivariable analysis of nine clinical characteristics in the SEER database.

	**One year**		**Three years**		**Five years**	
**Character**	**Hazard ratio (CI95)**	***P*-value**	**Hazard ratio (CI95)**	***P*-value**	**Hazard ratio (CI95)**	***P*-value**
Sex	0.98 (0.94–1.02)	0.241	NA (NA)	NA	0.94 (0.92–0.96)	<0.01
Lateral	NA (NA)	NA	1.38 (1.24–1.54)	<0.01	1.6 (1.47–1.75)	<0.01
T	1.47 (1.42–1.51)	<0.01	1.36 (1.33–1.38)	<0.01	1.28 (1.26–1.3)	<0.01
N	0.95 (0.9–1.01)	0.082	1.01 (0.98–1.05)	0.461	1.03 (1–1.06)	0.027
M	2.55 (2.43–2.67)	<0.01	2.67 (2.59–2.76)	<0.01	2.67 (2.6–2.75)	<0.01
Age	2.79 (2.68–2.91)	<0.01	1.94 (1.9–1.99)	<0.01	1.73 (1.7–1.77)	<0.01
LNR	1.35 (1.3–1.41)	<0.01	1.3 (1.26–1.34)	<0.01	1.28 (1.25–1.31)	<0.01
Grade	1.32 (1.27–1.37)	<0.01	1.18 (1.15–1.21)	<0.01	1.1 (1.08–1.13)	<0.01
Node_number	0.75 (0.72–0.79)	<0.01	NA(NA)	NA	1.1 (1.08–1.13)	<0.01

### Constructing Colon Cancer Prognostic Prediction Models Based on Molecular Features

Molecular markers have demonstrated potential power in the prognosis of colon cancer (Nosho et al., 2010; Carethers and Jung, 2015). Considering the close relationship between cancer and environmental factors, we focused on 512 RNA-seq datasets, 461 miRNA expression profiles, and 347 Infinium 450k methylation data sets in the TCGA database. A total of 2,492 differentially expressed lncRNAs, 2,967 differentially expressed mRNAs, 280 differentially expressed miRNAs, and 11,043 differentially expressed DNA methylation sites were identified in colon cancer samples compared with paracancerous control samples, as described in section “Materials and Methods.”

In the training set, 11, 7, and 6 lncRNAs showed a significant association with 1-, 3-, and 5-year survival, respectively, and the maximum classifiers AUC values were 0.788, 0.833, and 0.825, respectively (Supplementary Figures 4–6A,B); eight, eight, and four mRNAs showed a significant association with 1-, 3-, and 5-year survival and the AUC values were 0.793, 0.784, and 0.849, respectively (Supplementary Figures 7–9A,B); the miRNA numbers were 7, 10, and 9, and the AUC values were 0.826, 0.759, and 0.849, respectively (Supplementary Figures 10–12A,B). The DNA methylation sites were 5, 7, and 7, and the AUC values were 0.833, 0.894, and 0.876, respectively (Supplementary Figures 13–15A,B). The top three lncRNAs were AC133528, AC109927, and AL021707; the top three mRNAs were TMEM88B, GHRHR, and ZC3HAV1L; the top three miRNAs were hsa-mir-545, has-mir-548k, and hsa-mir-374a; and the top three DNA methylation sites were cg17863551, cg08491964, and cg04067612.

We further integrated different moleculars to construct prediction models due to molecular mutual regulation. In the training set, the combinations of molecular features consisting of 11, 13, and 11 features showed a significant association with 1-, 3-, and 5-year survival, respectively, and the maximum AUC values were 0.915, 0.884, and 0.869 (Supplementary Figures 16–18A). Among the molecular features, the most important were DNA methylation sites on cg01515427, cg03024587, and cg04067612. Of all the molecular models, “survivalROC” were achieved significant results (Supplementary Figures 16–18B), and the Kaplan–Meier survival curves showed significant difference for the training set and test set (Supplementary Figures 16–18C,E) (*p* < 0.05).

**FIGURE 1:**
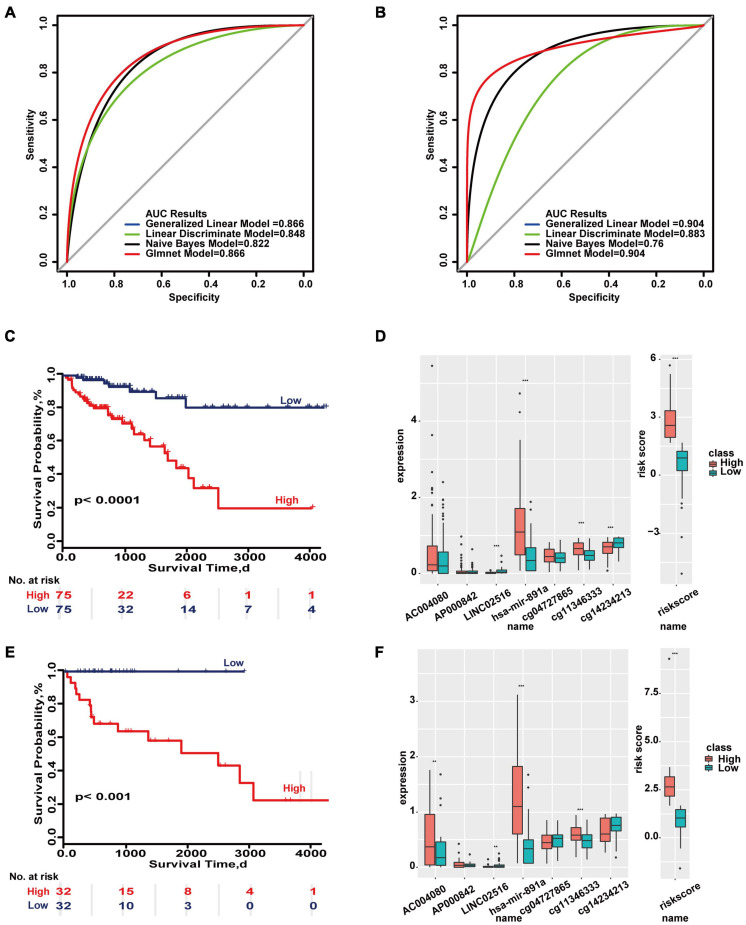
Important molecular features in overall survival based on the TCGA database. **(A)** ROC curves of the molecular-based overall prognosis model in the training set and test set. **(B)** Survival ROC curves in the two sets. **(C,E)** Kaplan–Meier survival curves of the two sets. **(D,F)** Distributions of molecular expressions in high- and low-risk groups of the two sets. **p < 0.05, ***p < 0.01.

This study also integrated all molecules to construct a molecular predictive model for the overall prognosis of colon cancer. The molecular colon cancer overall prognosis prediction model contains 6 molecules (AC004080, AP000842, LINC02516, hsa-mir-891a, cg04727865, cg14234213). The maximum AUC areas of the training set and test set are 0.866 and 0.904, respectively (Figures 1A–B). The Kaplan-Meier survival curves of the high and low risk groups in the training set and the test set were significantly different (*p* < 0.05).’

Even in some models, the expression of a single molecule showed no difference but the combination of all molecules showed significant differences in the two sets (Figures 1D,F). These results indicate there are differences in the prognosis of colon cancer at both clinical and molecular levels, and therefore carrying out stratified prognostic analysis of colon cancer is valuable.

### One-Year Prognostic Prediction Models Based on Both Molecular Features and Combinations of Clinical Characteristics

The relationship of the combinations of clinical characteristics and the molecular features were analyzed based on the survival time. The results showed that age ≥ 70&T3 and M0 were significantly different between the high- and low-risk groups of all molecular models in the two sets (Supplementary Figure 19). The following results indicate that combinations of clinical characteristics and molecular features may affect prognosis of colon cancer.

First, we developed a comprehensive prediction model based on the combination of clinical and molecular characteristics for 1-year survival. In the training set and test set, the maximum classifier AUC values were 0.713 and 0.825 based on a risk score that was calculated by five differential DNA methylation sites (cg17863551, cg01515427, cg01790269, cg14803765, cg15002294) and three combinations of clinical characteristics (age ≥ 70&T3, LNR median, T4&poorly differentiated, or undifferentiated) (Supplementary Figures 20A,B). “SurvivalROC” achieved AUC values of 0.868 and 0.819 in the two sets (Supplementary Figure 20C). The ability to evaluate the model can also be seen from the nomogram constructed by multivariable Cox regression analysis. According to the corresponding scores of each feature, if the cumulative score is less than 42, the survival probability at 1 year may be greater than 95% (Supplementary Figure 20D).

In the rest of the clinical and molecular composite models, a risk score was calculated by 11 lncRNAs (AC133528, AC097637, AL513327, LINC01675, AC018629, MIR31HG, AC008686, TSPEAR-AS2, AC125603, AC011603, AC119428) and four combinations of clinical factors (age < 70&moderately differentiated, LNR low&age < 70, LNR none&M0, LNR median&T3). AUC values were 0.805 and 0.763 in the two sets (Supplementary Figures 21A,B). The “survivalROC” values were 0.814 and 0.739 (Supplementary Figure 21C). If the cumulative score is less than 64, the 1-year survival probability will be greater than 95% according to the nomogram (Supplementary Figure 21D). For a risk score calculated by eight mRNAs (TMEM88B, PLCG2, PADI3, SH2D7, GABRD, PRSS1, RNF151, TMPRSS11E) and two combinations of clinical factors (age < 70&M0, LNR high), AUC values were 0.783 and 0.747 in two sets, and “survivalROC” values were 0.78 and 0.753. If the cumulative score is less than 28, the 1-year survival probability will be greater than 95% according to the nomogram (Supplementary Figures 22A–D). For a risk score calculated by seven miRNAs (hsa-mir-545, hsa-mir-3942, hsa-mir-641, hsa-mir-4632, hsa-mir-7641, hsa-mir-187, hsa-mir-3615) and two combinations of clinical factors (age ≥ 70&T3,M0), the AUC values were 0.786 and 0.72 in two sets. The “survival” ROC values were 0.746 and 0.739. If the cumulative score is less than 30, the 1-year probability will be greater than 95% according to the nomogram (Supplementary Figures 23A–D). The Kaplan–Meier survival curves were significantly different in training set and test set (*p* < 0.05) (Supplementary Figures 20–23E,G). The distribution of each combination of clinical characteristics and molecular feature can be seen from the heatmap, and the risk score is also significantly different (Supplementary Figures 20–23F,H).

**FIGURE 2:**
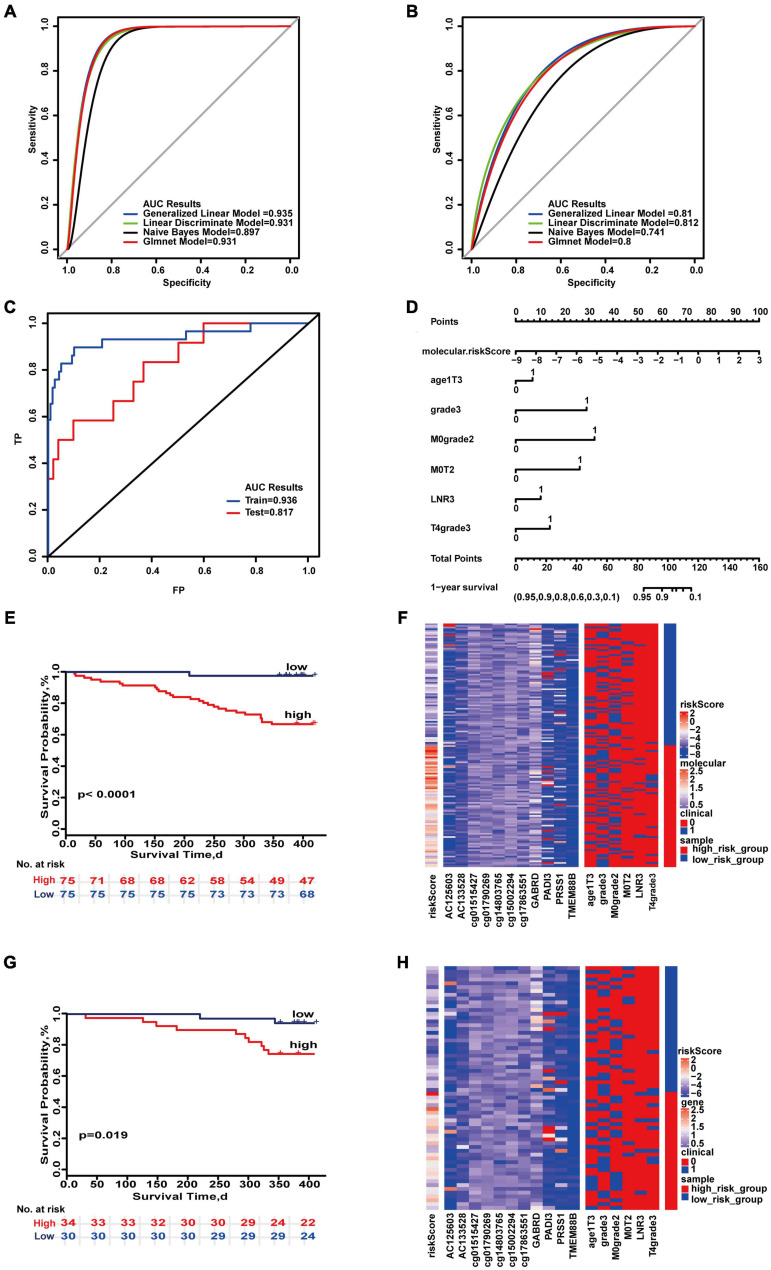
Important combinations of clinical characteristics and molecular features for 1-year survival time. **(A,B)** ROC curves of the training set and test set. **(C)** ROC survival curves of the two sets. **(D)** Nomogram of multivariate Cox regression based on molecular features and combinations of clinical characteristics. **(E,G)** Kaplan–Meier survival curves of the two sets. **(F,H)** Heat maps of the two sets used to compare the differences between high- and low-risk groups.

The 1-year prognostic prediction model was based on the risk scores composed of 11 molecules (AC125603, AC133528, cg01790269, cg14803765, cg15002294, cg17863551, cg01515427, GABRD, ADI3, PRSS1, TMEM88B) and six combinations of clinical characteristics (T4&poorly differentiated or undifferentiated, poorly differentiated or undifferentiated, M0&moderately differentiated, age ≥ 70&T3, M0&T2,LNR high). The maximum AUC values of the two sets were 0.935 and 0.812 (Figures 2A,B). “SurvivalROC” values were 0.936 and 0.817 (Figure 2C). From the nomogram, if the cumulative score is less than 84, the 1-year survival rate will be greater than 95% (Figure 2D). The c-index is 0.901 (95% CI, 0.843–0.960). The Kaplan–Meier survival curves of the high- and low-risk groups were significantly different (*p* < 0.05) (Figures 2E,G), and the distribution of the expression of the high- and low-risk groups composed of clinical and molecular was also significantly different in the heat maps of two sets (Figures 2F,H). For 1-year survival, the most important feature was the molecular risk score, and the most important clinical combination feature is T4&poorly differentiated or undifferentiated.

### Mining of Eight Clinical Combination Characteristics and 13 Molecular Features From 3-Year Prognostic Prediction Models

Similar to the strategy of constructing the 1-year prognostic prediction model, the 3-year prognostic prediction model shows the complement of molecular features and clinical characteristics in predicting prognosis. Risk scores were calculated from seven methylation sites (cg03024587, cg03265268, cg03957898, cg04031361, cg04067612, cg0891964, cg13279566) and 11 combinations of clinical features (well differentiated, M0&age∘< 70, M0&T1, M0&T2, M0&T4, M1&T3, M0, LNR 0&age < 70, LNR 0&T3&age < 70, LNR median, T3&age ≥ 70). The maximum AUC values of the training set and test set were 0.839 and 0.783, respectively. “SurvivalROC” achieved AUC values of 0.83 and 0.84 (Supplementary Figures 24A–C). In accordance with each feature, if the cumulative score is less than 150, the patient’s 3-year survival rate is greater than 80% (Supplementary Figure 24D). The Kaplan–Meier survival curves were significantly different in the training set and test set (*p* < 0.05) (Supplementary Figures 24E,G). The distribution of the combination of clinical and molecular features can be seen from the heat map (Supplementary Figures 24F,H).

The risk score was calculated using seven lncRNAs (AC002091, AC025211, AC109927, AL159972, AL356124, LINC01807, PTPRJ-AS1) and nine combinations of clinical features (poorly differentiated or undifferentiated, M0&age < 70, M0&T2, M0T4, M1&T3, M0, LNR 0&age < 70, LNR median&age ≥ 70, LNR median), and the maximum AUC values in the two sets were 0.795 and 0.674. “SurvivalROC” values were 0.794 and 0.693 (Supplementary Figures 25A–C). According to the nomogram, if the cumulative score is less than 72, the 3-year survival rate will be greater than 95% (Supplementary Figure 25D). The Kaplan–Meier survival curves between the high- and low-risk groups were significantly different (*p* < 0.05) (Supplementary Figures 25E,G). The distribution of the expression of each combination of clinical features and molecules can be seen from the heat map; the risk scores significantly different in high- and low-risk groups (Supplementary Figures 25F,H).

The risk score was calculated using eight mRNAs (AP3B2, ATP2B3, CD300LG, DNAH14, GHRHR, OR1J4, PLGG2, SLC28A2) and five combinations of clinical characteristics (M0&T1, LNR 0&age < 70, LNR 0&T3&age < 70, LNR median, T4&poorly differentiated or undifferentiated), and the maximum AUC areas of the training set and test set were 0.747 and 0.751, respectively. “SurvivalROC” values were 0.77 and 0.744. If the cumulative score is less than 66, the 3-year survival rate will be greater than 95% (Supplementary Figures 26A–D). Kaplan–Meier survival curves of the high- and low-risk groups were significantly different (*p* < 0.05) (Supplementary Figures 26E,G). The expressions of each combination of clinical features and molecule were significantly different in risk score in the heat map (Supplementary Figures 26F,H).

The risk score was calculated using 10 miRNAs (hsa-miR-2114, hsa-miR-3926, hsa-miR-5001, hsa-miR-5091, hsa-miR-545, hsa-miR-548k, hsa-miR-605, hsa-miR-641, hsa-miR-6798, hsa-miR-765) and nine combinations of clinical features (poorly differentiated or undifferentiated, M0&age < 70, M0&T1, M0&T2, LNR 0&age < 70, LNR 0&M0, LNR median&T3, LNR high, T4&poorly differentiated or undifferentiated), and the maximum AUC values in the training set and test set were 0.804 and 0.744, respectively. “SurvivalROC” values were 0.804 and 0.716 (Supplementary Figures 27A–C). If the cumulative score is less than 92, the 3-year survival rate will be greater than 80% (Supplementary Figure 27D). The Kaplan–Meier survival curves were significantly different in the two sets (*p* < 0.05) (Supplementary Figures 27E,G). From the heat map, the expression of each combination of clinical feature and molecule was significantly different in risk score (Supplementary Figures 27F,H).

**FIGURE 3:**
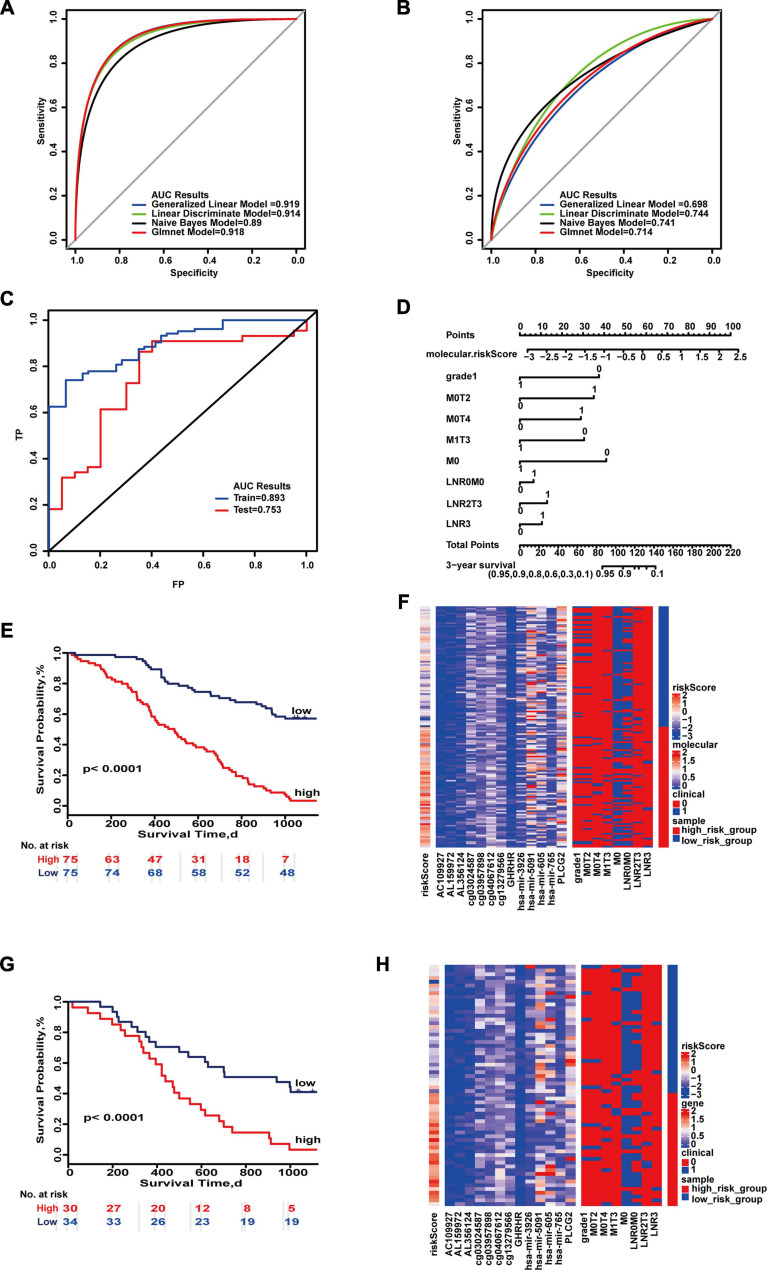
Important combinations of clinical characteristics and molecular features for 3-year survival time. **(A,B)** ROC curves of the training set and test set. **(C)** ROC survival curves of the two sets. **(D)** Nomogram of multivariate Cox regression based on molecular features and combinations of clinical characteristics. **(E,G)** Kaplan–Meier survival curves of the two sets. **(F,H)** Heat maps of the two sets used to compare the differences between high- and low-risk groups.

Finally, the 3-year prognostic prediction model was composed of 13 molecules (AC109927, AL159972, AL356124, cg03957898, cg04067612, cg13279566, cg03024587, GABRD, PLCG2, hsa-miR-3926, hsa-miR-5091, hsa-miR-605, hsa-miR-765) and eight combinations of clinical characteristics (M0, M0&T4, LNR 0&M0, M0&T2, M1&T3, LNR median&T3, LNR high, poorly differentiated, or undifferentiated). The maximum AUC values of the training set and test set were 0.919 and 0.744 (95% CI, 0.594–0.867) (Figures 3A,B). “SurvivalROC” values were 0.893 and 0.753, respectively (Figure 3C). As seen on the nomogram, if the cumulative score is less than 82, the 3-year survival rate will be greater than 95% (Figure 3D). The *c*-index is 0.773 (95% CI, 0.728–0.819). The Kaplan–Meier survival curves were significantly different in the high- and low-risk groups (*p* < 0.05) (Figures 3E,G), and the expressions of clinical and molecular combinations were also significantly different (Figures 3F,H). For 3-year survival, the most important feature is the risk score, and the most important clinical combination feature is M0.

### Evaluation of 5-Year Survival in Colon Cancer Using Three Combinations of Clinical Characteristics and 11 Molecular Features

**FIGURE 4:**
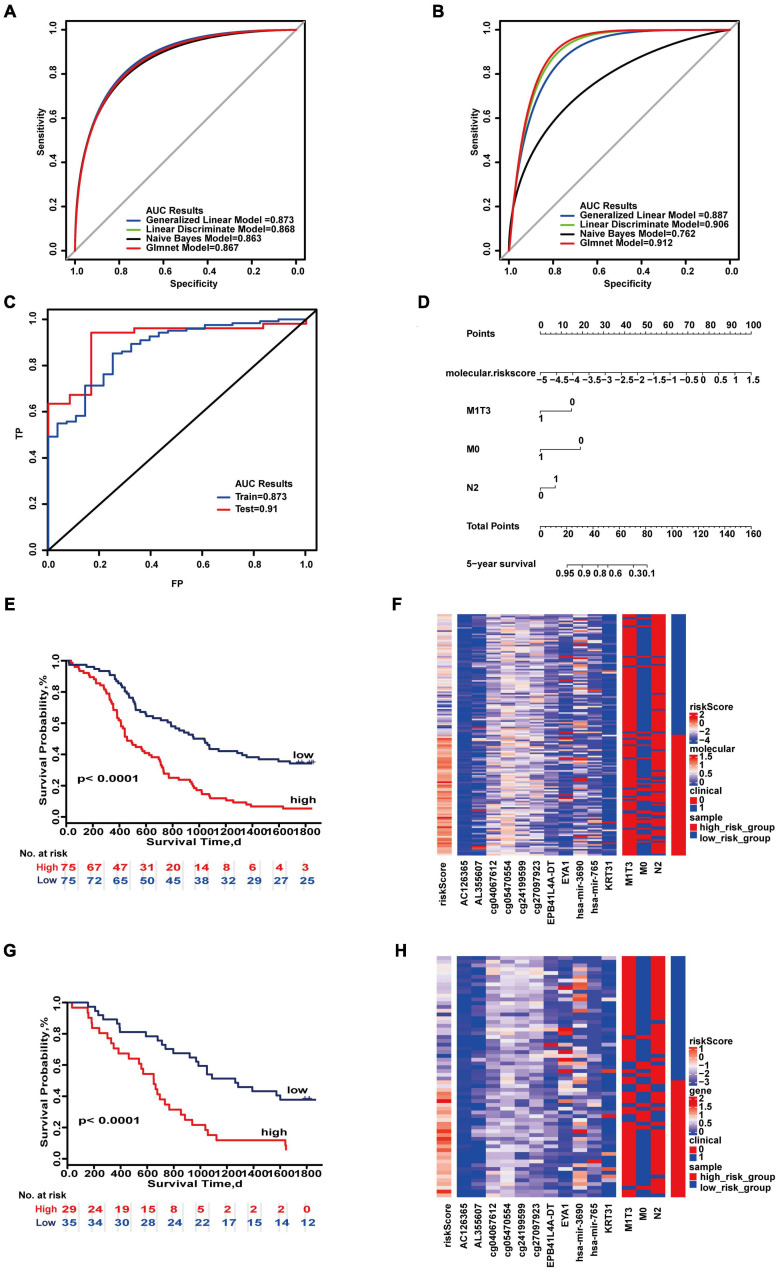
Important combinations of clinical characteristics and molecular features for 5-year survival time. **(A,B)** ROC curves of the training set and test set. **(C)** ROC survival curves of the two sets. **(D)** Nomogram of multivariate Cox regression based on molecular features and combinations of clinical characteristics. **(E,G)** Kaplan–Meier survival curves of the two sets. **(F,H)** Heat maps of the two sets used to compare the differences between high- and low-risk groups.

We also evaluated the impact of different molecular and clinical features on the 5-year survival of patients (Supplementary Figures 28A–H, 29A–H, 30A–H, 31A–H). Molecular features from these four categories may be used to evaluate prognosis of colon cancer. The 5-year prognostic prediction model analysis for the combination of integrated molecular and clinical features was composed of 11 molecules (AC126365, AL355607, cg05470554, cg24199599, cg27097923, cg04067612, EPB41L4A-DT, EYA1, KRT31, hsa-miR-3690, hsa-miR-765) and three combinations of clinical characteristics (M0, M1&T3, N2). The 5-year prognostic prediction model analysis for the combination of integrated molecular and clinical features was composed of 11 molecules (AC126365, AL355607, cg05470554, cg24199599, cg27097923, cg04067612, EPB41L4A-DT, EYA1, KRT31, hsa-miR-3690, hsa-miR-765) and three combinations of clinical characteristics (M0, M1&T3, N2). The maximum AUC values of the training set and test set were 0.873 and 0.912, respectively (Figures 4A,B). “SurvivalROC” values were 0.873 and 0.91 (Figure 4C). From the nomogram, if the cumulative score is less than 16, the 5-year survival rate will be greater than 95% (Figure 4D). The c-index is 0.718 (95% CI, 0.671–0.765). Kaplan–Meier survival curves of the high- and low-risk groups were significantly different (*p* < 0.05) (Figures 4E,G), and the expression of clinical and molecular groups was also significantly different (Figures 4F,H). The most important feature is the risk score, and M0 is the most important clinical combination feature for 5-year survival.

### Constructing Multi-Type Colon Cancer Prediction Models With Survival Time

**FIGURE 5:**
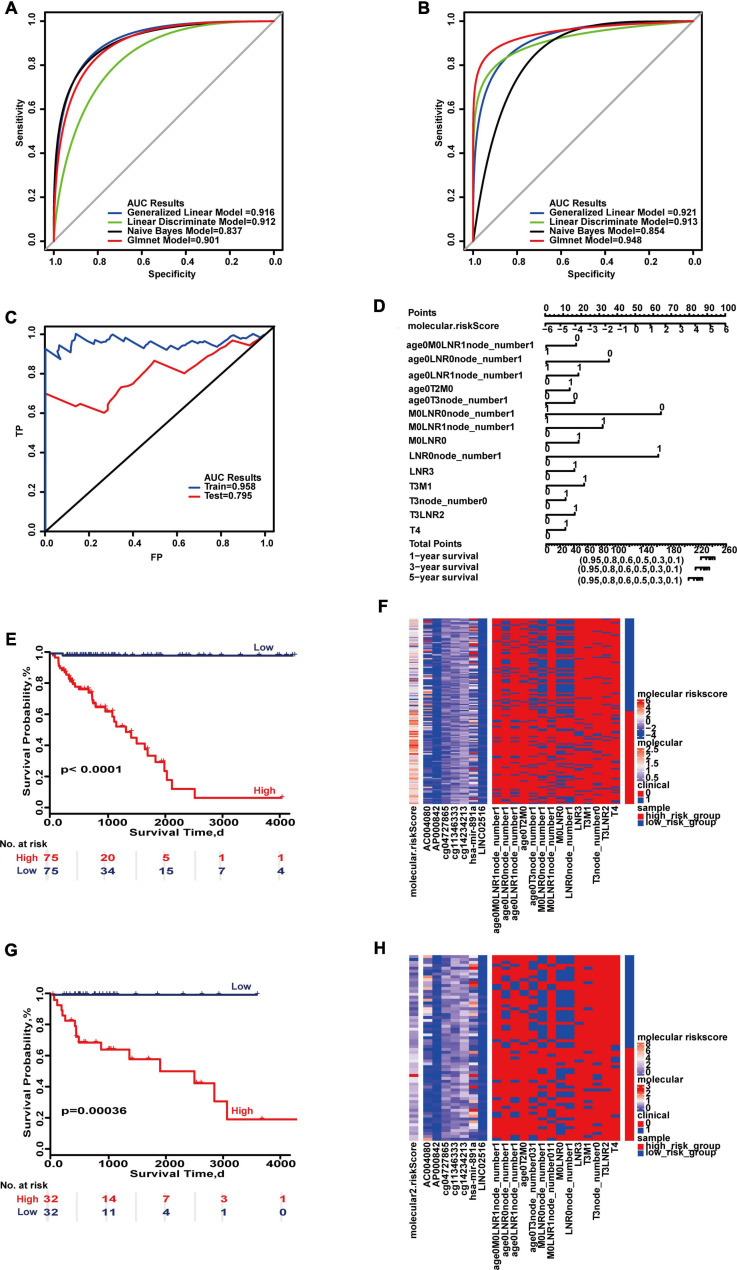
Important combinations of clinical characteristics and molecular features for overall survival. **(A,B)** ROC curves of the training set and test set. **(C)** ROC survival curves of the two sets. **(D)** Nomogram of multivariate Cox regression based on molecular features and combinations of clinical characteristics. **(E,G)** Kaplan–Meier survival curves of the two sets. **(F,H)** Heat maps of the two sets used to compare the differences between high- and low-risk groups.

Multi-type overall survival prediction models for all colon cancer patients were also developed to take into account follow-up times (Supplementary Figures 32–39). The results showed that maximum AUC reached 0.916 and 0.948 in the training set and test set (Figures 5A,B). “SurvivalROC” values were 0.958 and 0.795, respectively, and the *c*-index is 0.921 (95% CI, 0.872–0.971) (Figure 5C). If the cumulative score is less than 224, the survival probability of 1 year may be greater than 95%; for 3 years, the cumulative score is less than 212, and for 5 years the cumulative score is less than 202 (Figure 5D). The survival curves of the high- and low-risk score groups were significantly different (Figures 5E,G), and the heat maps displayed the difference of the survival time model (Figures 5F,H).

## Discussion

The survival time of postoperative patients with colon cancer shows significant difference among patients. This leads to great difficulty in determining treatment decisions in clinical practice. This study showed that the prediction model may provide a tool for prognostic evaluation. For example, there may be a prognostic factor for a specific survival rate, and there may be a poor prognostic factor for patients whose lifetime is less than 1 year. Survival for more than 5 years is referred to as clinical cure. In this study, several prognostic prediction models for 1-, 3-, and 5-year survival time were determined based on a large number of samples with clinical information from SEER and clinical and molecular characteristics in TCGA. These models provide convenience for guiding clinical personalized treatment and can provide a strong treatment plan for patients with poor prognosis to improve the patient survival rate. Maximum AUC values were more than 0.8 by molecular and clinical features, which means great improvement of the prognostic prediction effect. In each predictive model, a nomogram was provided to determine the survival probability. For example, if the cumulative score is less than 84, the 1-year survival rate will be greater than 95%. The factor for 1-year prediction was based on six combinations of clinical characteristics (T4&poorly differentiated or undifferentiated, poorly differentiated or undifferentiated, M0&moderately differentiated, age ≥ 70&T3, M0&T2,LNR high) and a risk score based on a total of 11 molecular features (cg01515427, AC125603, AC133528, cg01790269, cg14803765, cg15002294, cg17863551, GABRD, PADI3, PRSS1, and TMEM88B). The factor for 3-year survival was based on eight combinations of clinical characteristics (M0, M0&T4, LNR none&M0, M0&T2, M1&T3, LNR median&T3, LNR high, poorly differentiated, or undifferentiated) and 13 molecular features (cg03024587, AC109927, AL159972, AL356124, cg03957898, cg04067612, cg13279566, GABRD, PLCG2, hsa-mir-3926, hsa-mir-5091, hsa-mir-605, and hsa-mir-765). The factor for 5-year survival was based on three combinations of clinical characteristics (M0, M1&T3, LNR median) and 11 molecular features (cg04067612, AC126365, AL355607, cg05470554, cg24199599, cg27097923, EPB41L4A-DT, EYA1, KRT31, hsa-mir-3690, and hsa-mir-765). Thus, the prognostic prediction of colon cancer is a complex process. Our analysis demonstrates the feasibility of combining molecular features and combinations of clinical characteristics for prognostic prediction of colon cancer patients.

Molecular factors play an important prognostic role in various cancers, and among these molecules, DNA methylation sites of the gene contribute the most power (Bird, 2002). For example, Huang et al. (2013) reported that ZIC1 promoter hypermethylation correlates with poor progression-free survival of ovarian cancer, and methylation of the ZIC1 gene, a putative tumor suppressor, may be a novel determinant of ovarian cancer outcome. Many molecular features in this study have been shown to be associated with the prognosis of colon cancer and are closely related to clinical pathological characteristics, For example, studies have found that miRNA-641 expression is strongly correlated with lymph node metastasis and stage in colon cancer (Yao et al., 2018).

In our study, we also focused on LNR. LNR is an important factor for prognostic prediction, but no cutoff threshold for LNR has been established. For example, Shinto et al. (2019) used the Akaike information criterion to categorize LNR by cutoffs of 0.16 and 0.22. Berger et al. used LNR quartiles to categorize LNR (LNR: < 0.05, 0.05–0.19, 0.2–0.39, and 0.4–1.0) (Berger et al., 2005). Therefore, an information gain method was developed to redefine the thresholds in this study. The thresholds were 0, 0.2, and 0.6 for 1 and 3 years and the thresholds were 0, 0.3, and 0.7 for 5 years. Our study also incorporated LNR into clinical factors to establish survival time. The impact of LNR on survival is more important and more effective than the N stage. Our results showed that the N stage was not significant in 1- and 3-year survival by multivariable Cox regression analysis. Therefore, we propose that LNR can replace N or that LNR should be added to TNM staging.

## Conclusion

The study demonstrates that models such as these are in general reliable. The prediction model based on a combination of both clinical characteristics and molecular features may be suitable for the evaluation of specific survival probability in colon cancer.

## Data Availability Statement

The original contributions presented in the study are included in the article/Supplementary Material, further inquiries can be directed to the corresponding author/s.

## Author Contributions

LW and YZ conceived the whole study. MS and MZ performed all data acquisition, programming, and code execution. HZ carried out the sample collection. HW and DZ carried out the data analysis. WX developed the methodology and created the data prediction model. HL was responsible for the data analysis and validation of the conclusion of the manuscript. JX and CG were responsible for the inspection of the manuscript. All authors have read and approved the final manuscript and contributed to the work presented in this manuscript.

## Conflict of Interest

The authors declare that the research was conducted in the absence of any commercial or financial relationships that could be construed as a potential conflict of interest.

## Footnotes

1 https://seer.cancer.gov/

2 https://www.cancer.gov/

3 https://xenabrowser.net/datapages/

4 https://CRAN.R-project.org/package=caret

5 http://www.r-project.org
