# Supplementary material for: Accurate Prediction of Prognosis by Integrating Clinical and Molecular Characteristics in Colon Cancer
Source: Front Cell Dev Biol. 2021 May 21;9:664415. doi: 10.3389/fcell.2021.664415 (PMC8176021; doi:10.3389/fcell.2021.664415)
Supplement: Supplementary file 1 [file Data_Sheet_1.docx]

**Accurate prediction of hierarchy prognosis integrated clinical and molecular**

**characteristic in colon cancer**


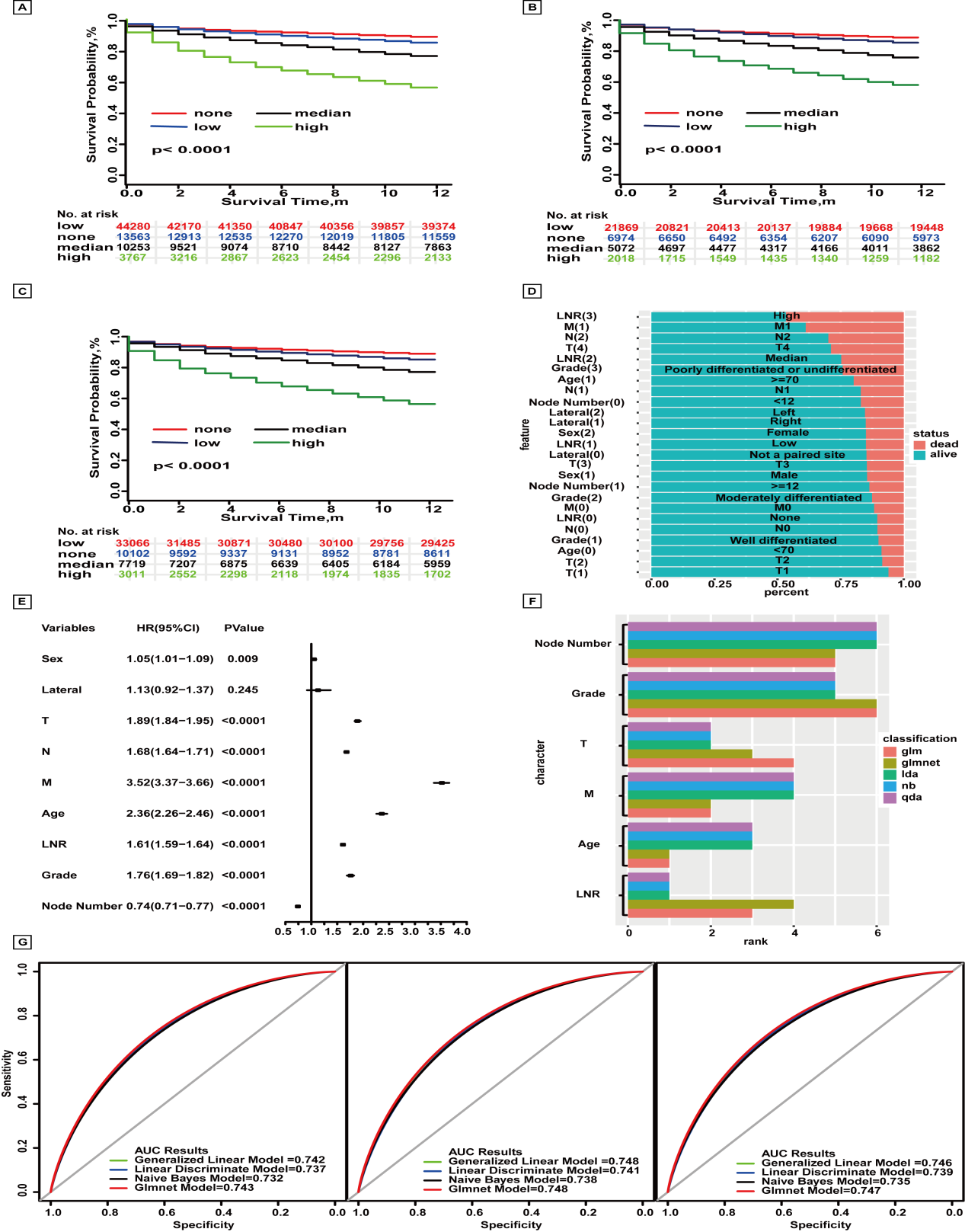


Supplementary Figure1.Prognostic prediction model of clinical characteristics based on SEER database for 1-year survival time . (A-C) Kaplan-Meier survival curves of the training set, test set and additional test set based on LNR. (D) Bar graphs of survival and death rates according to clinical characteristics. (E) Forest maps of univariable Cox analysis. (F) Ranking of important clinical characteristics. (G) ROC survival curves of the three sets based on combinations of clinical characteristics.


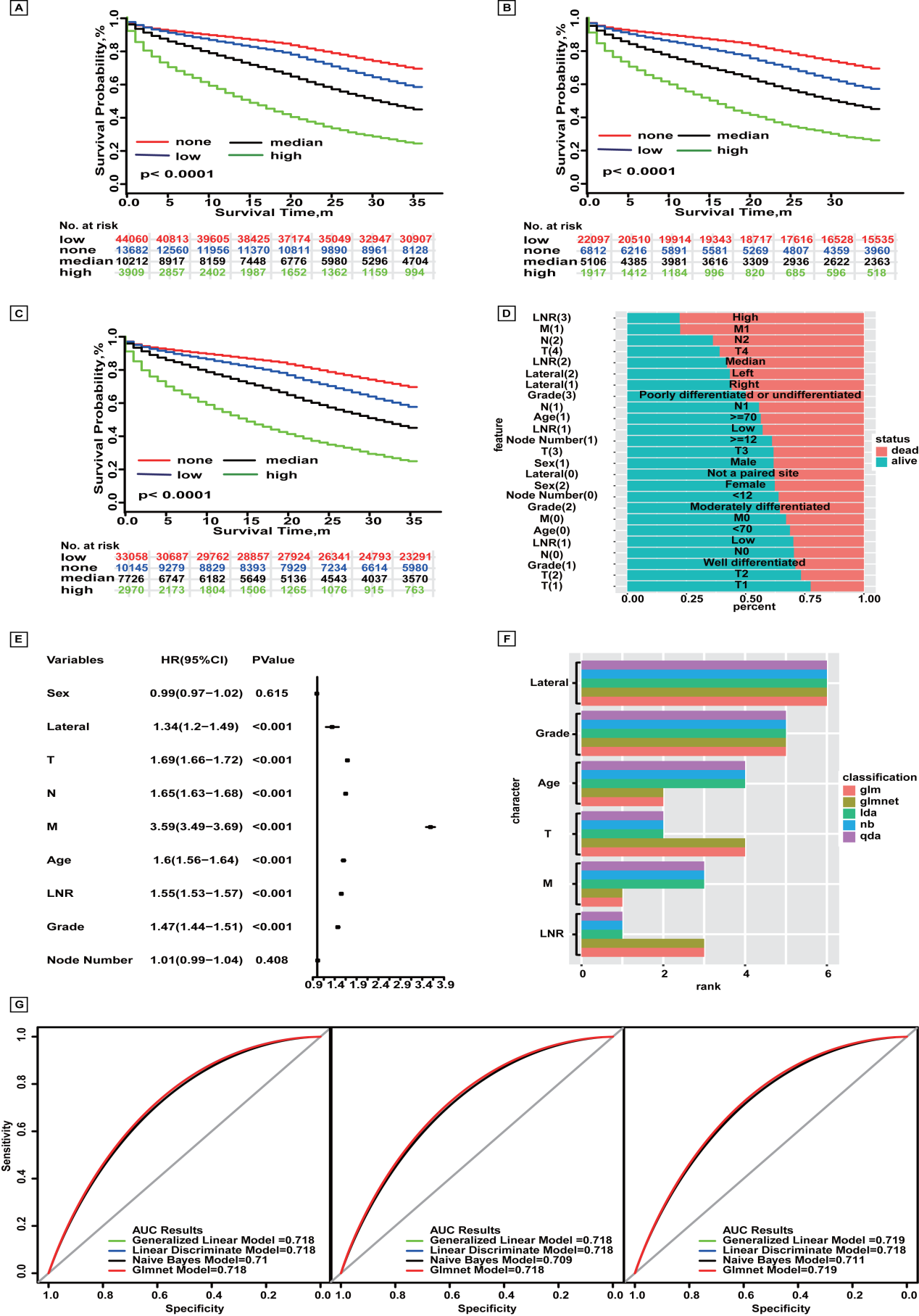


Supplementary Figure2. Prognostic prediction model of clinical characteristics based on SEER database for 3-year survival time. (A-C) Kaplan-Meier survival curves of the training set, test set and additional test set based on LNR . (D) Bar graphs of survival and death rates according to clinical characteristics. (E) Forest maps of univariable Cox analysis. (F) Ranking of important clinical characteristics.  (G) ROC survival curves of the three sets based on combinations of clinical characteristics.


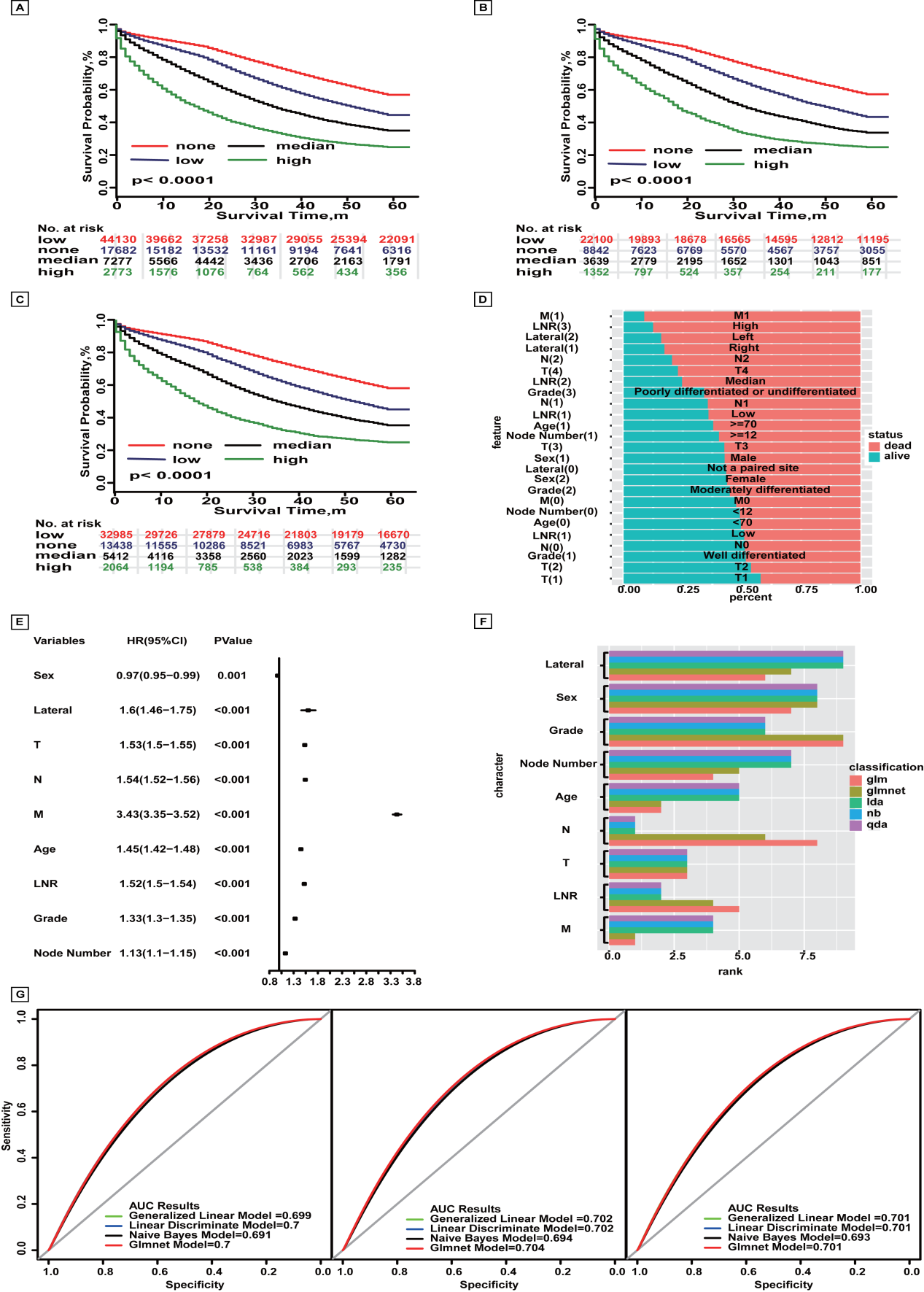


Supplementary Figure3. Prognostic prediction model of clinical characteristics based on SEER database for 5-year survival time. (A-C) Kaplan-Meier survival curves of the training set, test set and additional test set based on LNR. (D) Bar graphs of survival and death rates according to clinical characteristics. (E) Forest maps of univariable Cox analysis. (F)Ranking of important clinical characteristics. (G) ROC survival curves of the three sets based on combinations of clinical characteristics.


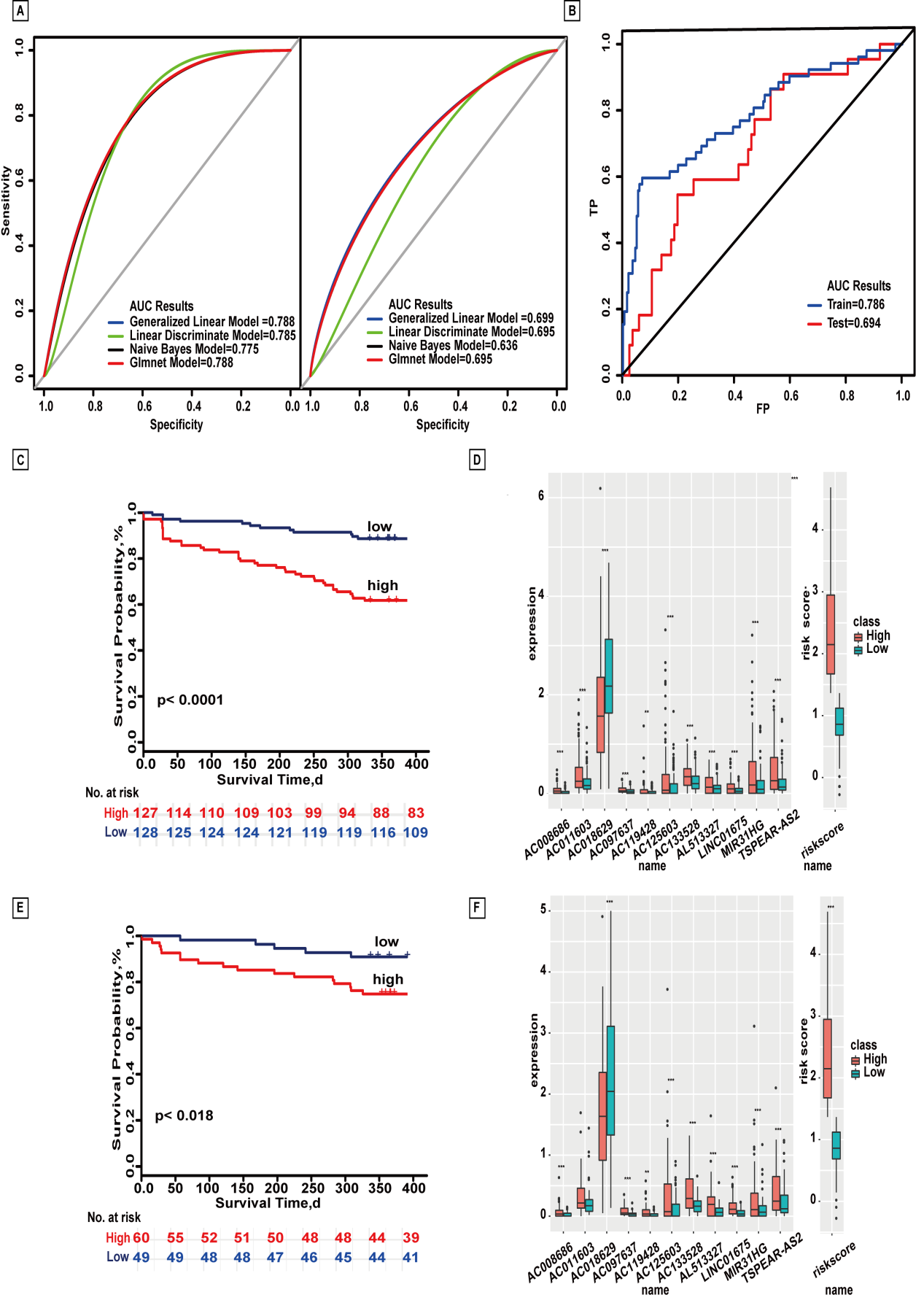


Supplementary Figure4. Important lncRNA features for 1-year survival based on TCGA database. (A) ROC curves of the training set and test set.(B) ROC survival curves of the two sets. (C,E)Kaplan-Meier survival curves of the two sets.(D,F) Distribution of lncRNA expression in high- and low-risk groups of the two sets. ***means p<0.01,**means p<0.05.


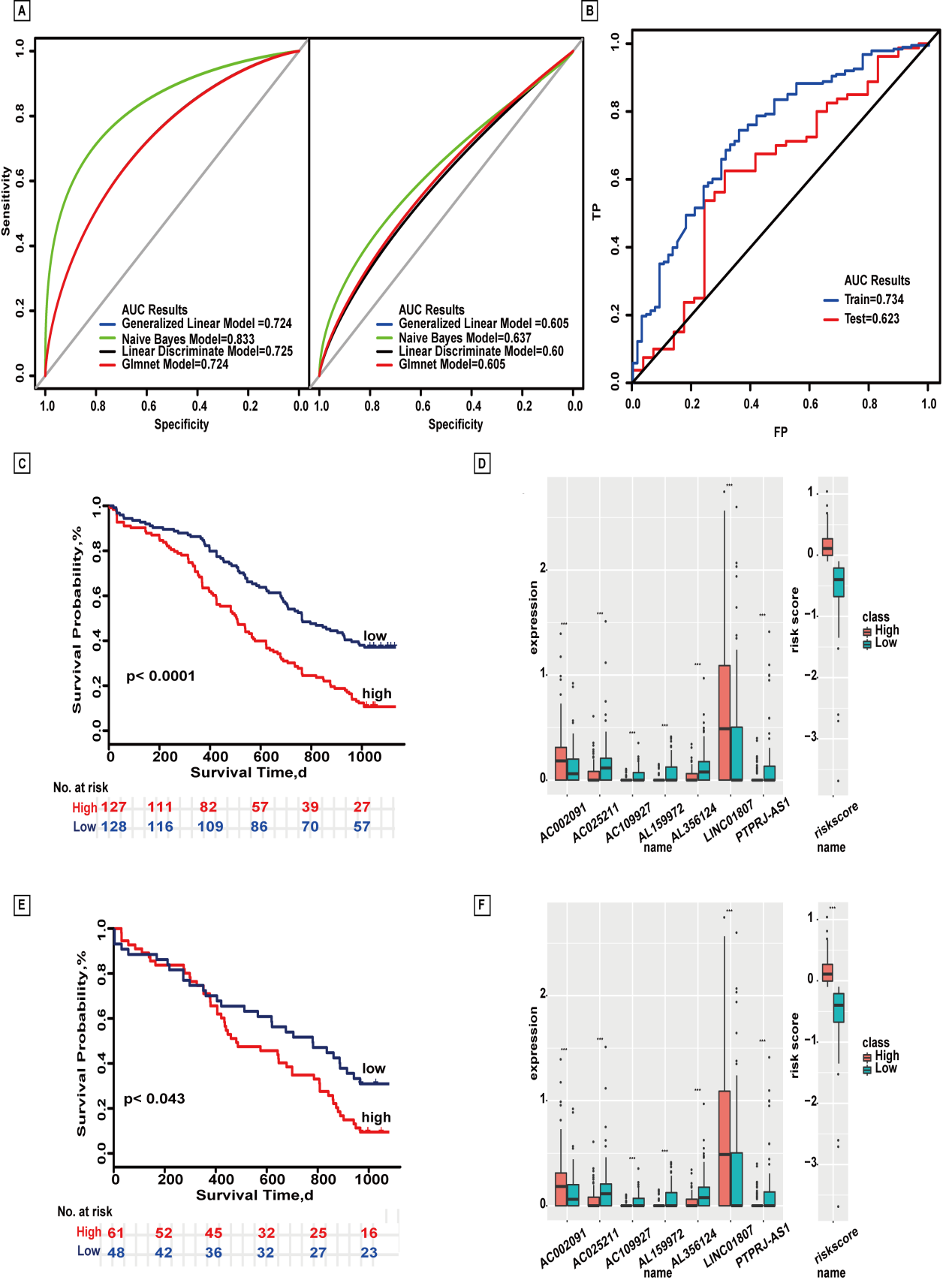


Supplementary Figure5. Important lncRNA features for 3-year survival based on TCGA database. (A) ROC curves of the training set and test set.(B) ROC survival curves of the two sets. (C,E)Kaplan-Meier survival curves of the two sets .(D,F) Distribution of lncRNA expression in high- and low-risk groups of the two sets. ***means p<0.01,**means p<0.05


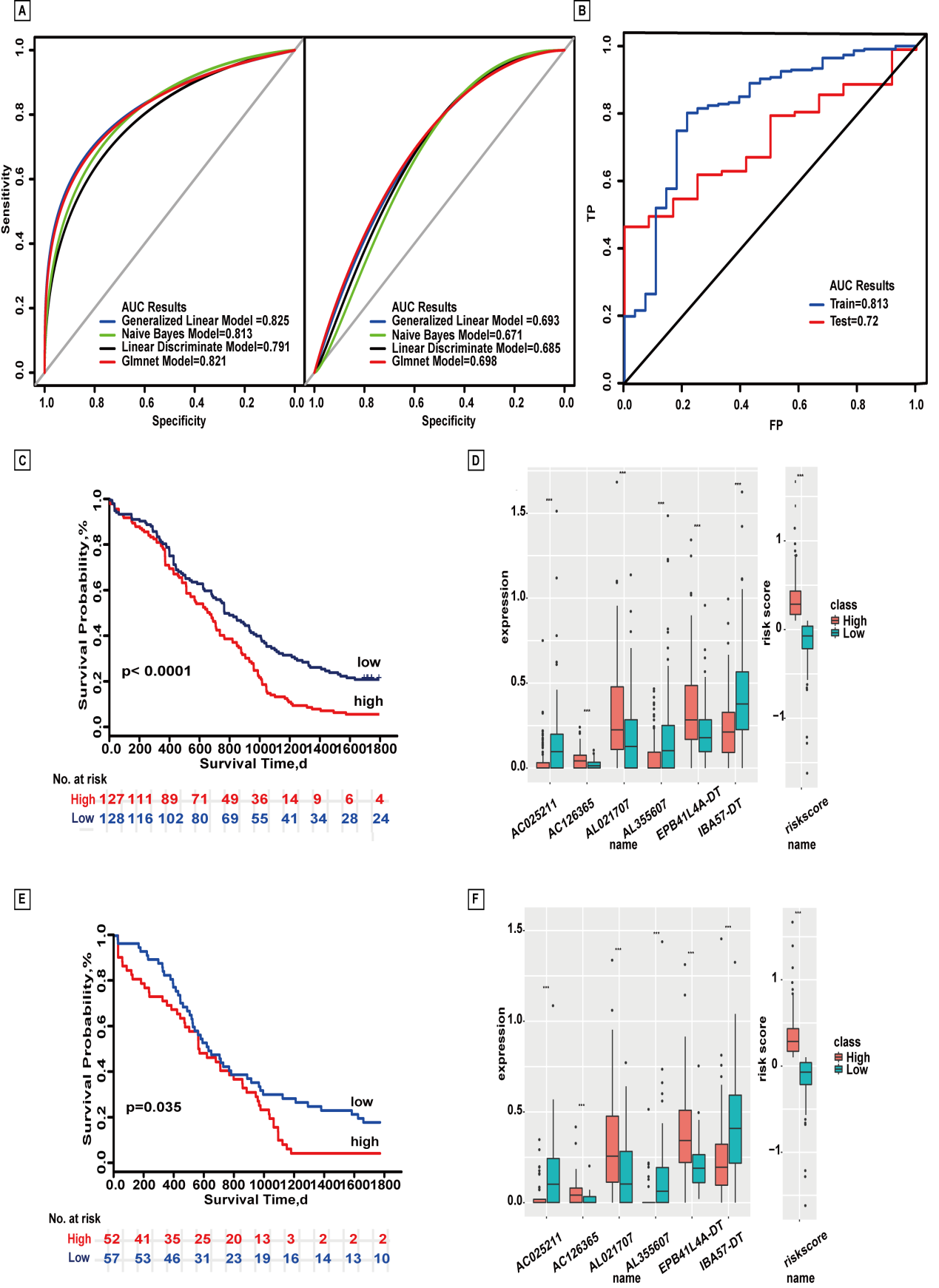


Supplementary Figure6. Important lncRNA features for 5-year survival based on TCGA database. (A) ROC curves of the training set and test set. (B) ROC survival curves of the two sets. (C,E)Kaplan-Meier survival curves of the two sets. (D,F) Distribution of lncRNA expression in high- and low-risk groups of the two sets. ***means p<0.01,**means p<0.05


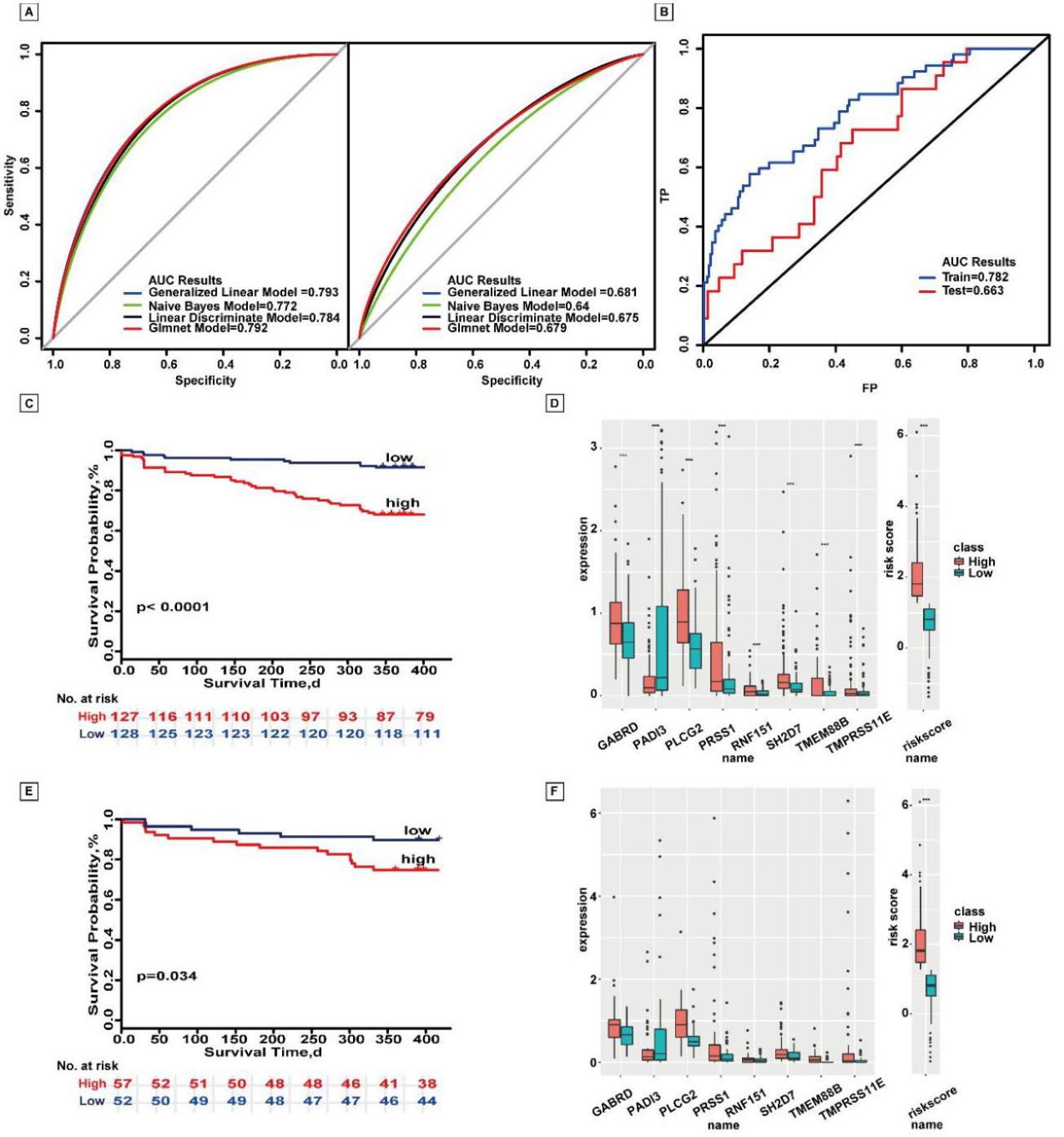


Supplementary Figure7. Important mRNA features for 1-year survival based on TCGA database . (A) ROC curves of the training set and test set. (B) ROC survival curves of the two sets. (C,E) Kaplan-Meier survival curves of the two sets. (D,F) Distribution of lncRNA expression in high- and low-risk groups of the two sets. ***means p<0.01,**means p<0.05.


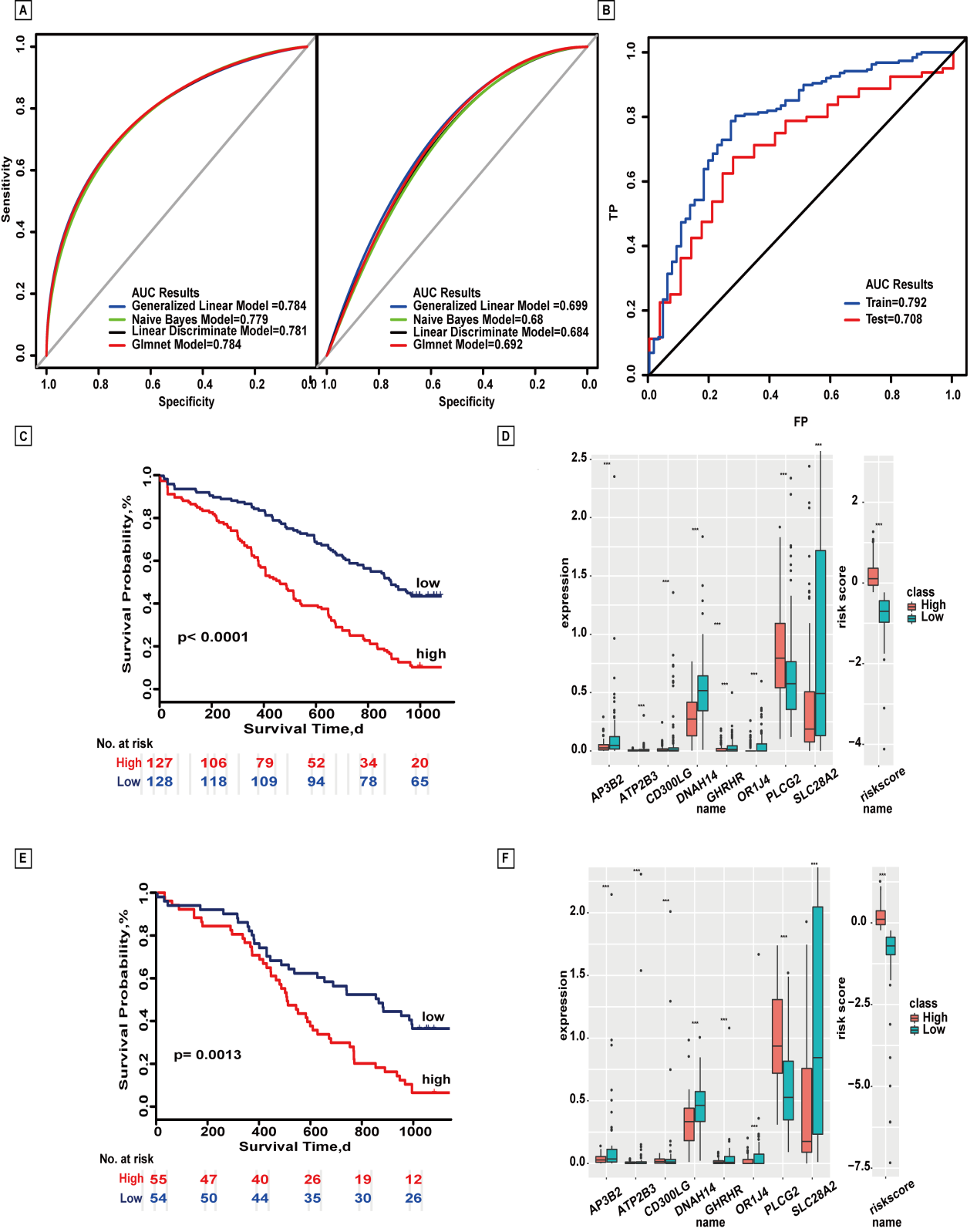


Supplementary Figure8. Important mRNA features for 3-year survival based on TCGA database. (A) ROC curves of the training set and test set. (B) ROC survival curves of the two sets. (C,E)Kaplan-Meier survival curves of the two sets. (D,F) Distribution of lncRNA expression in high- and low-risk groups of the two sets. ***means p<0.01,**means p<0.05.


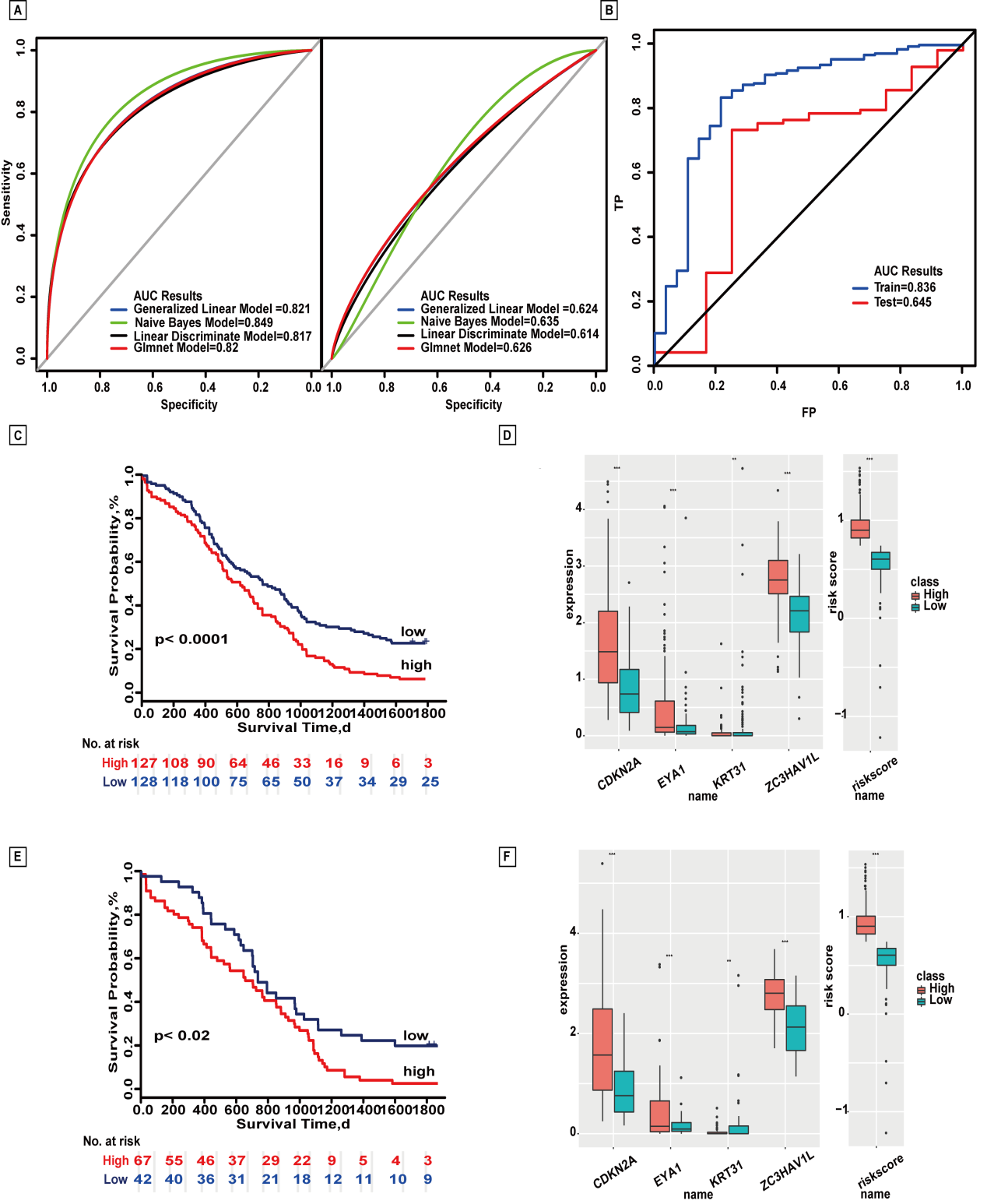


Supplementary Figure9. Important mRNA features for 5-year survival based on TCGA database. (A) ROC curves of the training set and test set. (B) ROC survival curves of the two sets. (C,E) Kaplan-Meier survival curves of the two sets. (D,F) Distribution of lncRNA expression in high- and low-risk groups of the two sets. ***means p<0.01,**means p<0.05.


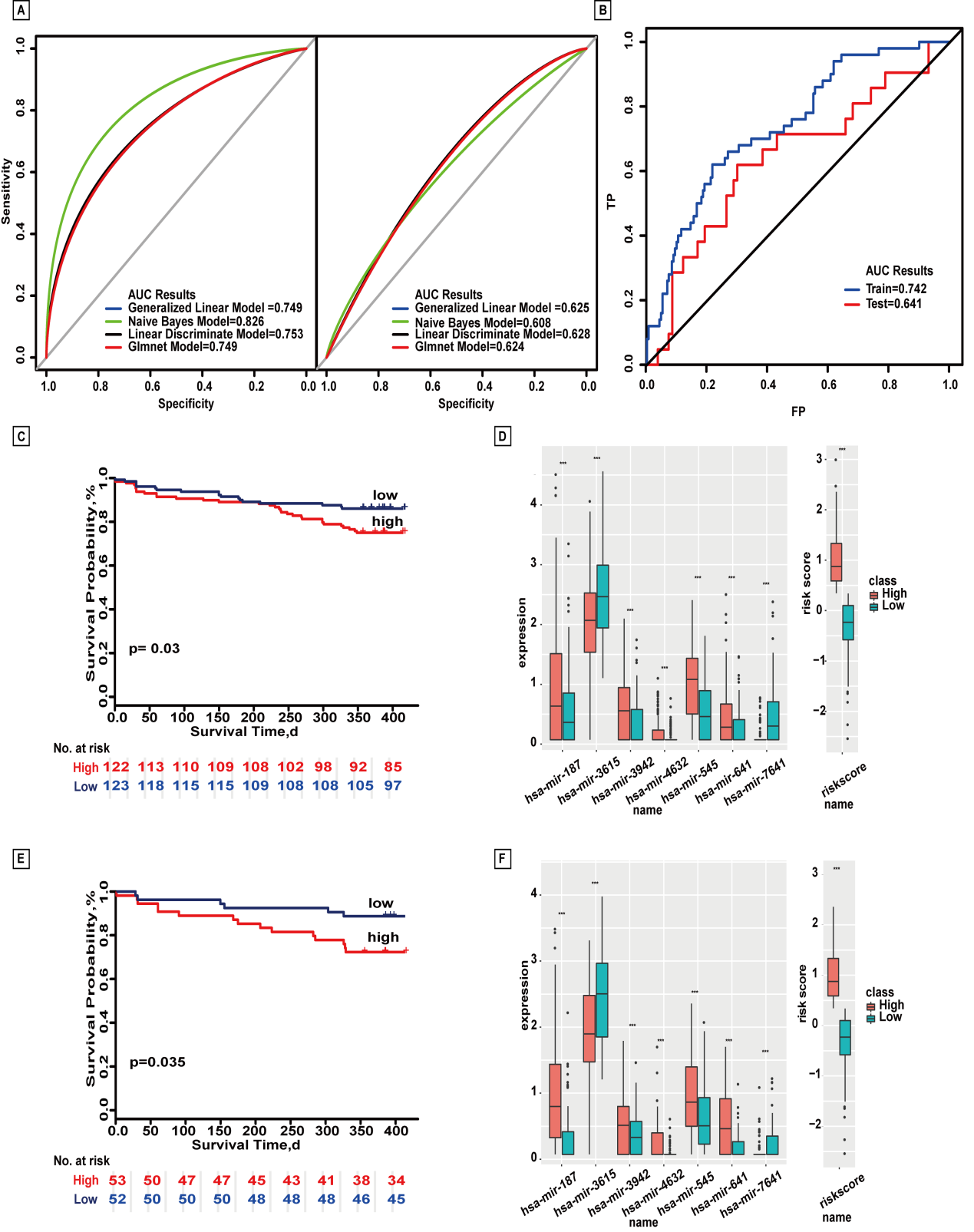


Supplementary Figure10. Important miRNA features for 1-year survival based on TCGA database. (A) ROC curves of the training set and test set. (B) ROC survival curves of the two sets. (C,E) Kaplan-Meier survival curves of the two sets. (D,F) Distribution of lncRNA expression in high- and low-risk groups of the two sets. ***means p<0.01,**means p<0.05.


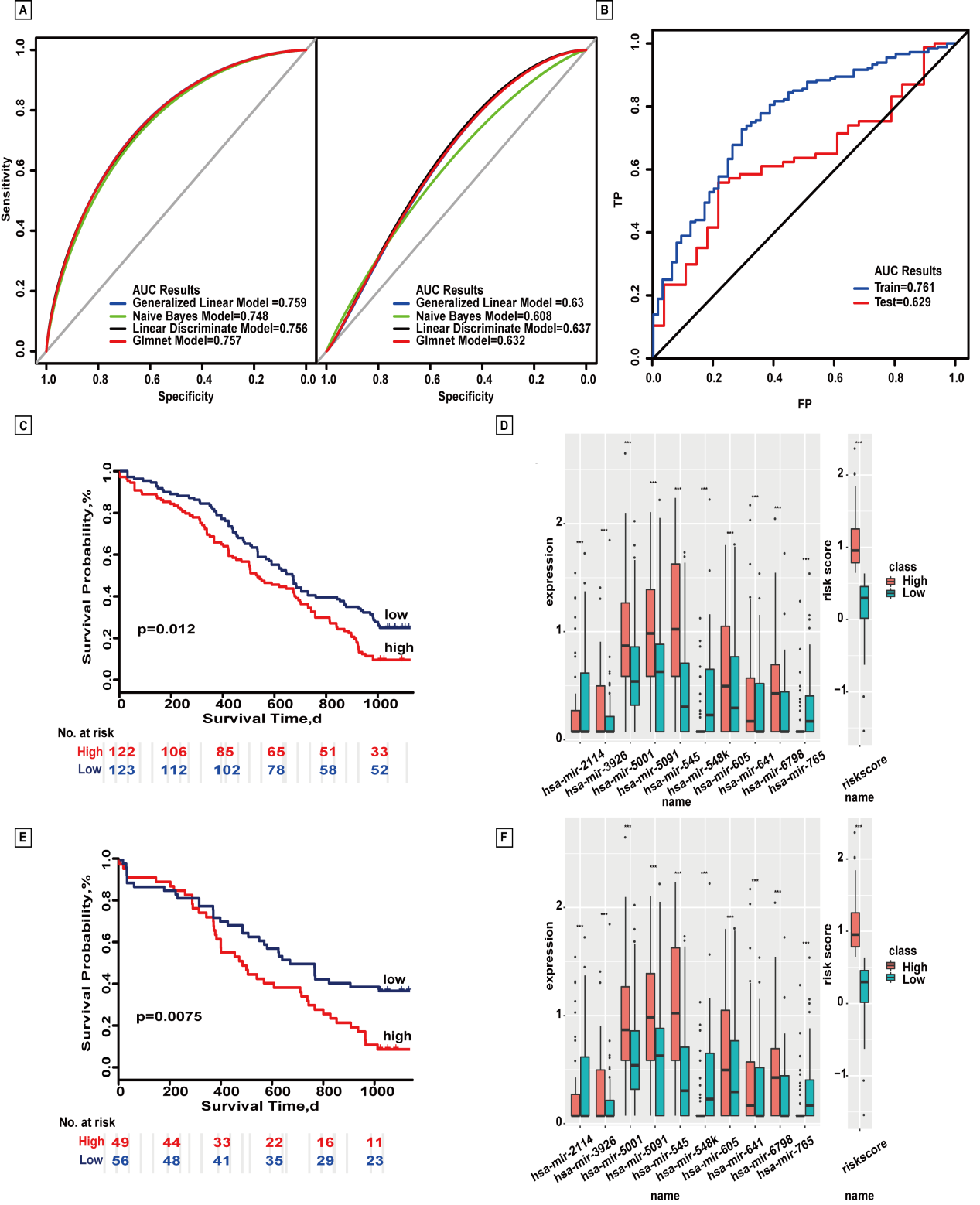


Supplementary Figure11. Important mRNA features for 3-year survival based on TCGA database. (A) ROC curves of the training set and test set.(B) ROC survival curves of the two sets.(C,E) Kaplan-Meier survival curves of the two sets. (D,F) Distribution of lncRNA expression in high- and low-risk groups of the two sets. ***means p<0.01,**means p<0.05.


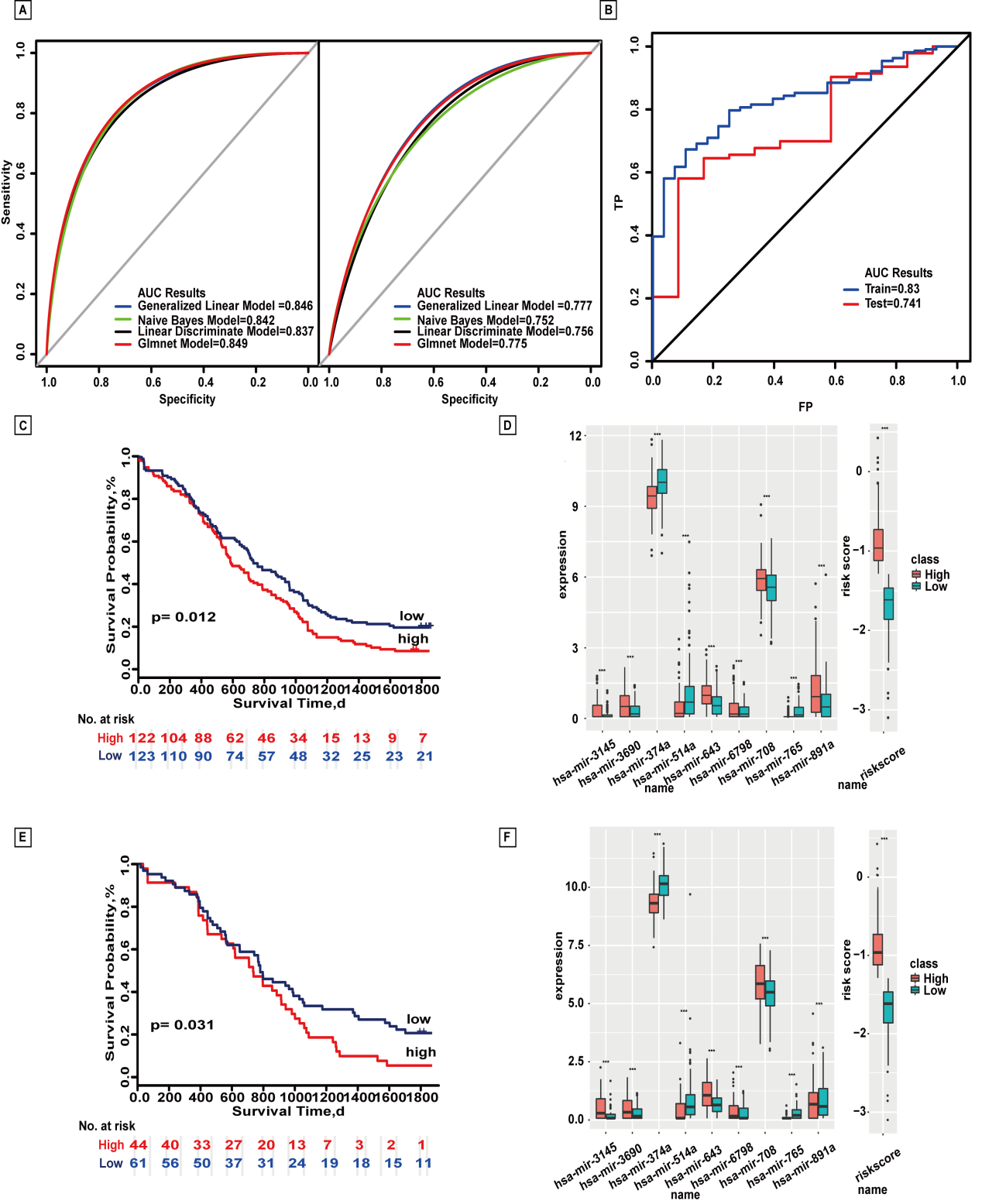


Supplementary Figure12. Important mRNA features for 5-year survival based on TCGA database. (A)ROC curves of the training set and test set.(B) ROC survival curves of the two sets. (C,E) Kaplan-Meier survival curves of the two sets . (D,F) Distribution of lncRNA expression in high- and low-risk groups of the two sets. ***means p<0.01,**means p<0.05.


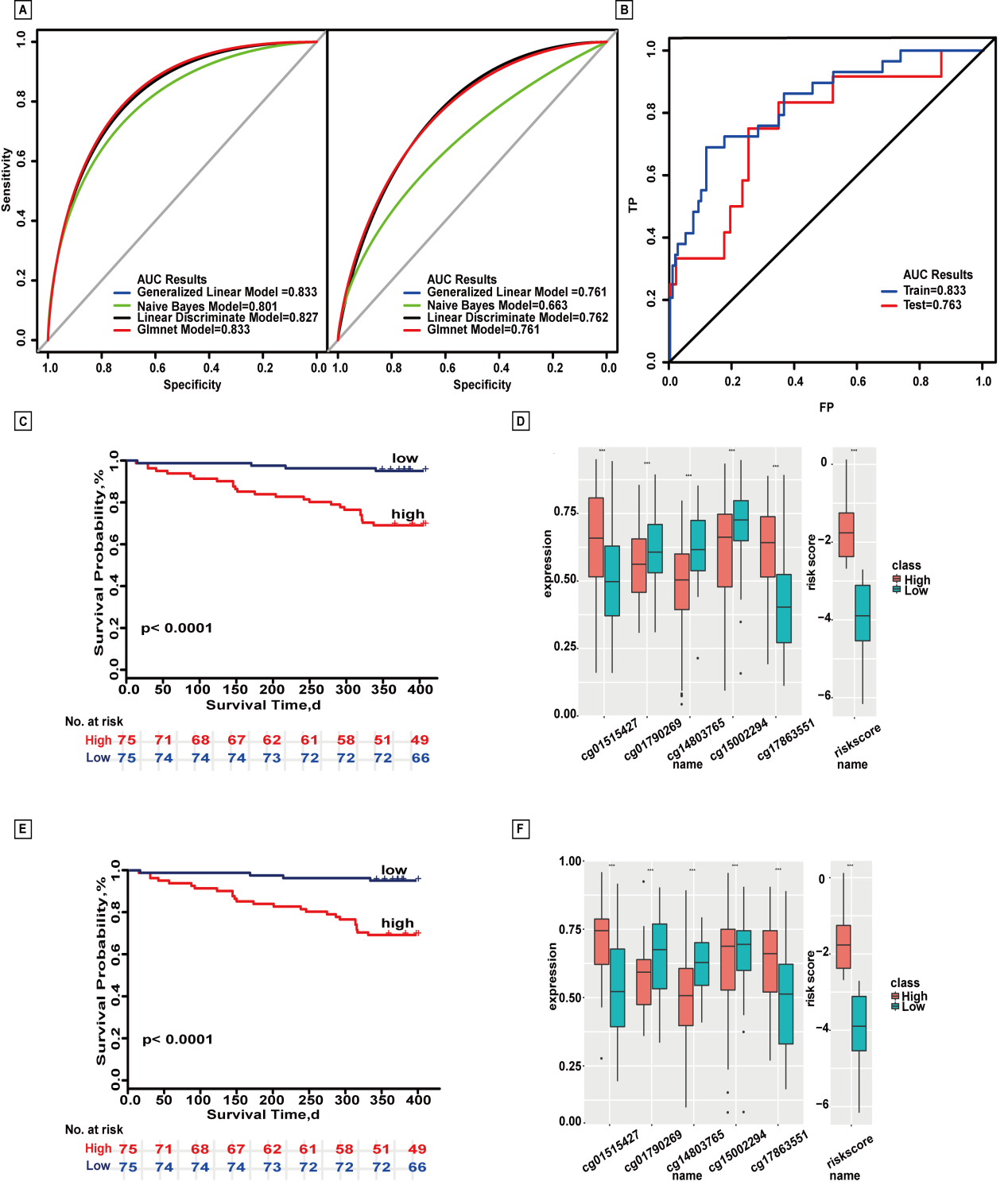


Supplementary Figure13. Important DNA methylation site features for 1-year survival based on TCGA database. (A) ROC curves of the training set and test set. (B) ROC survival curves of the two sets. (C,E) Kaplan-Meier survival curves of the two sets.(D,F) Distribution of lncRNA expression in high- and low-risk groups of the two sets. ***means p<0.01,**means p<0.05.


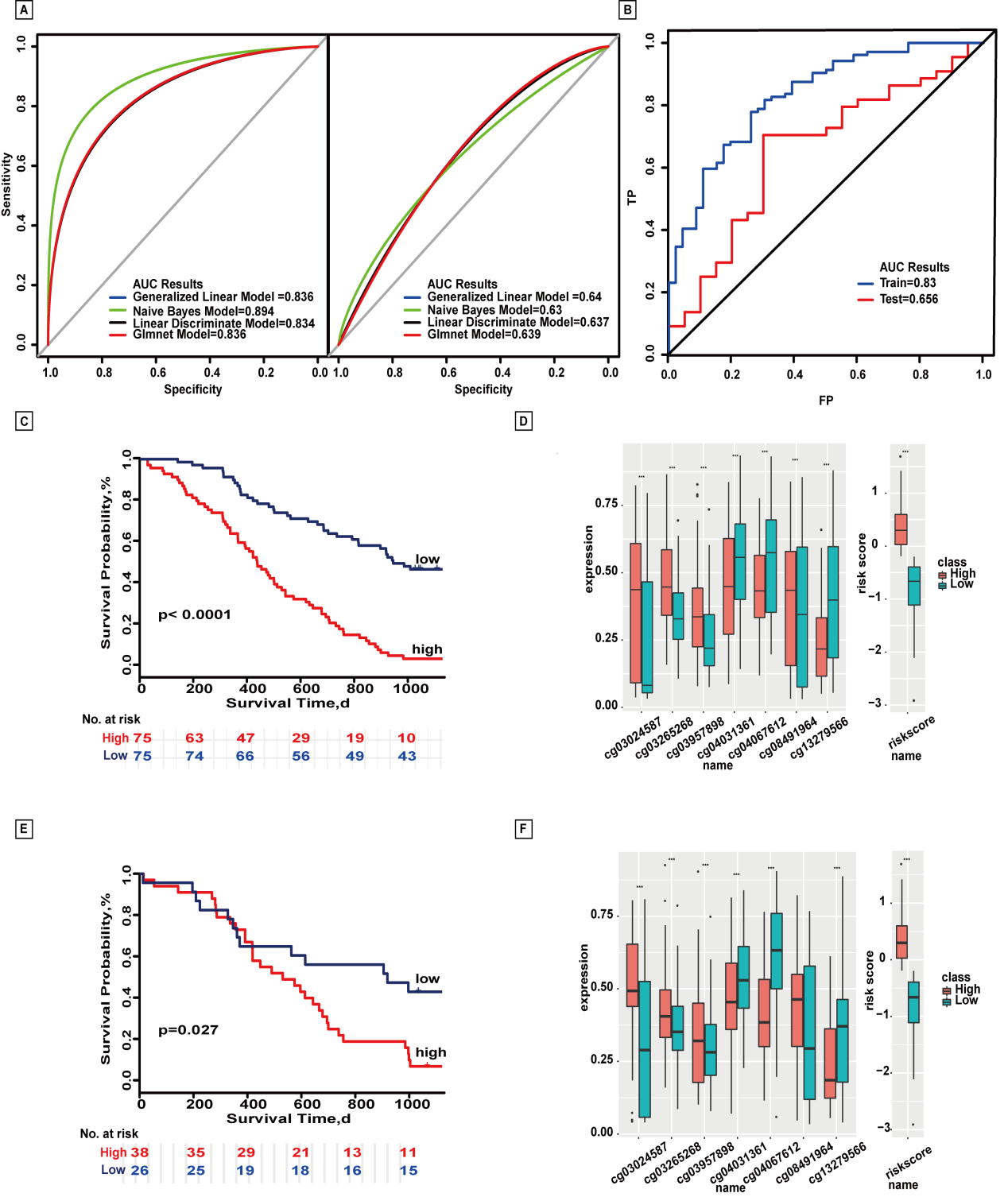


Supplementary Figure14. Important DNA methylation site features for 3-year survival based on TCGA database. (A) ROC curves of the training set and test set. (B) ROC survival curves of the two sets. (C,E) Kaplan-Meier survival curves of the two sets.(D,F) Distribution of lncRNA expression in high- and low-risk groups of the two sets. ***means p<0.01,**means p<0.05.


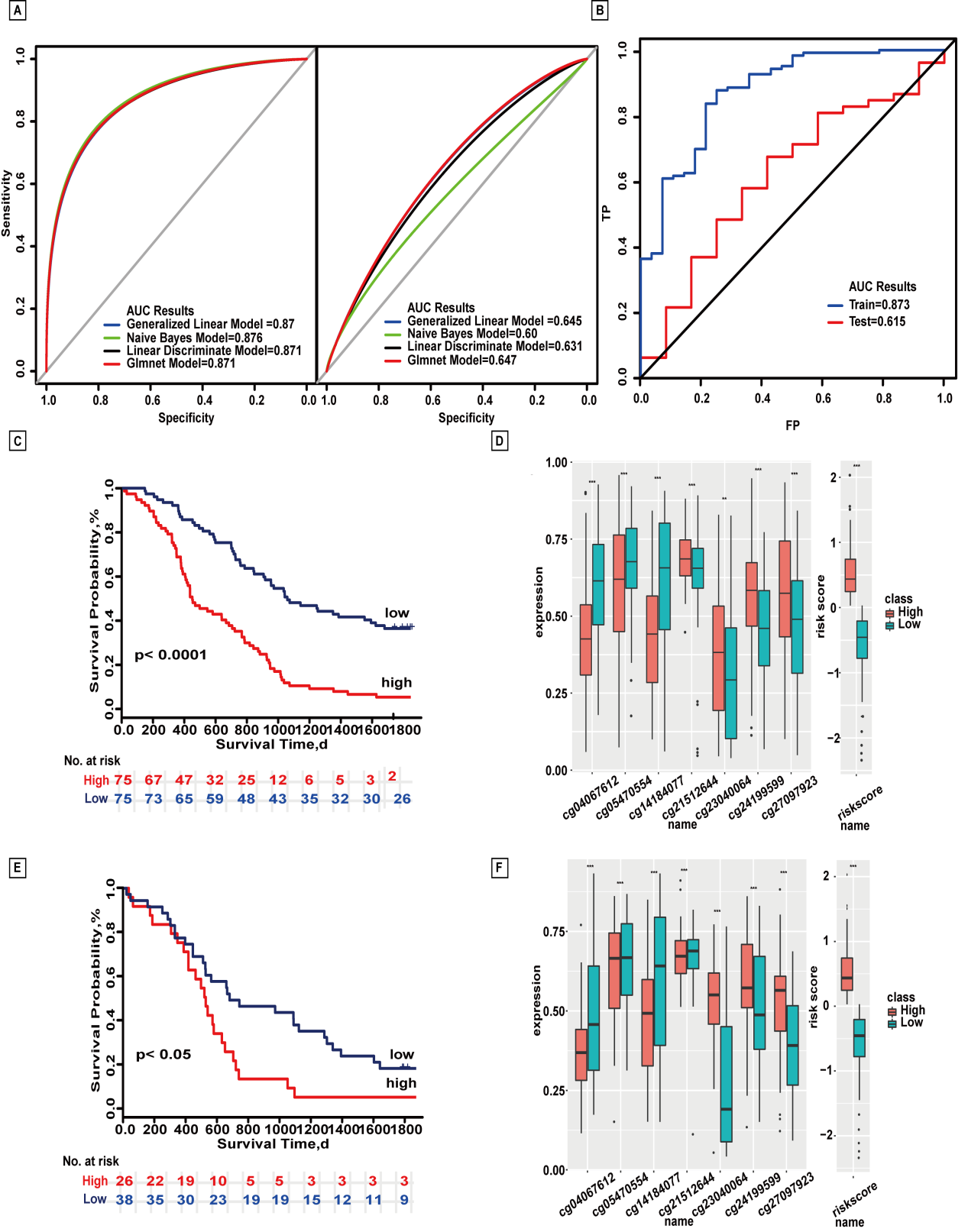


Supplementary Figure15. Important DNA methylation site features for 5-year survival based on TCGA database. (A) ROC curves of the training set and test set. (B) ROC survival curves of the two sets. (C,E) Kaplan-Meier survival curves of the two sets.(D,F)Distribution of lncRNA expression in high- and low-risk groups of the two sets. ***means p<0.01,**means p<0.05.


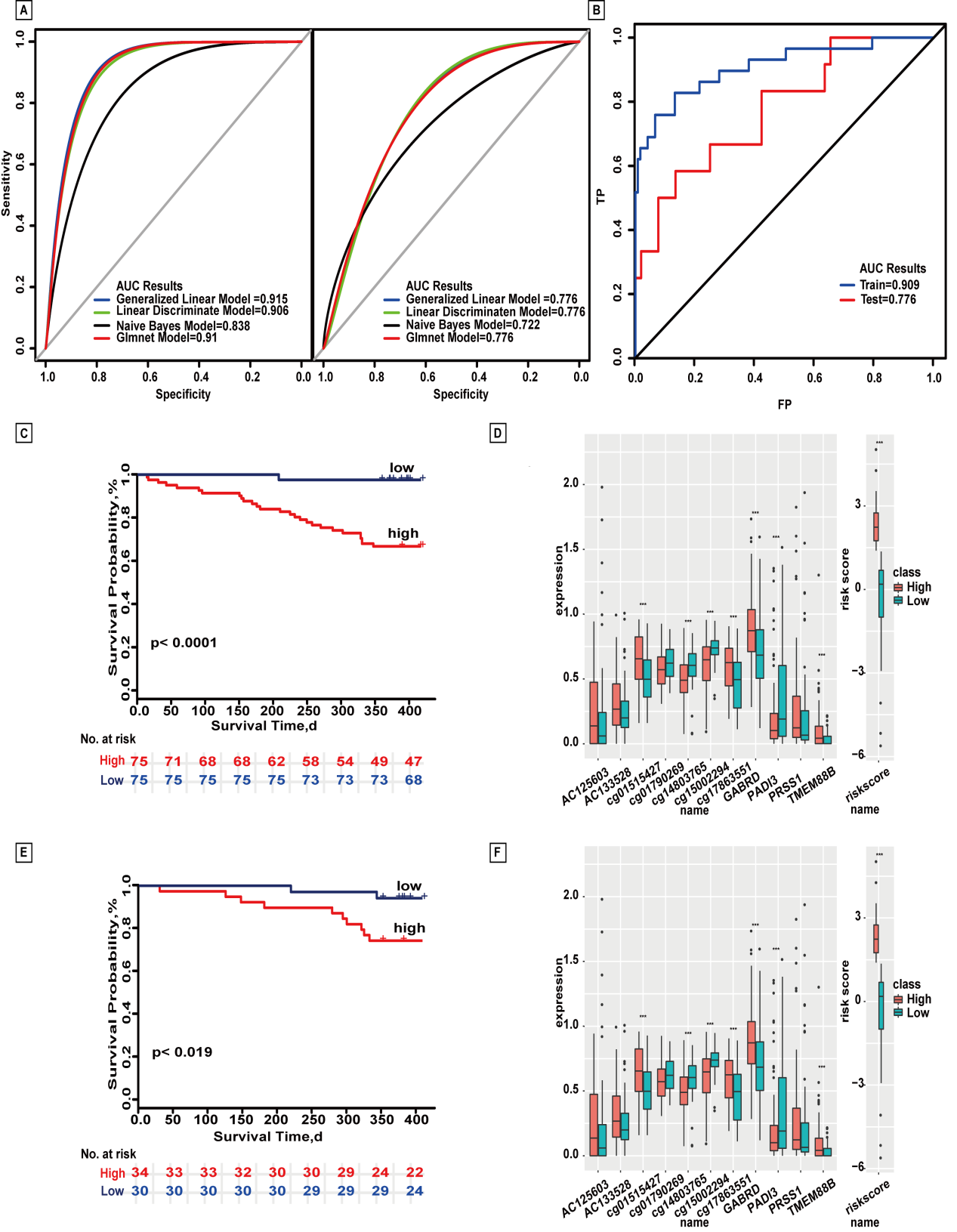


Supplementary Figure16. Important molecular features for 1-year survival based on TCGA database. (A) ROC curves of the training set and test set. (B) ROC survival curves of the two sets. (C,E) Kaplan-Meier survival curves of the two sets. (D,F) Distribution of molecular expression in high- and low-risk groups of the two sets. ***means p<0.01,**means p<0.05.
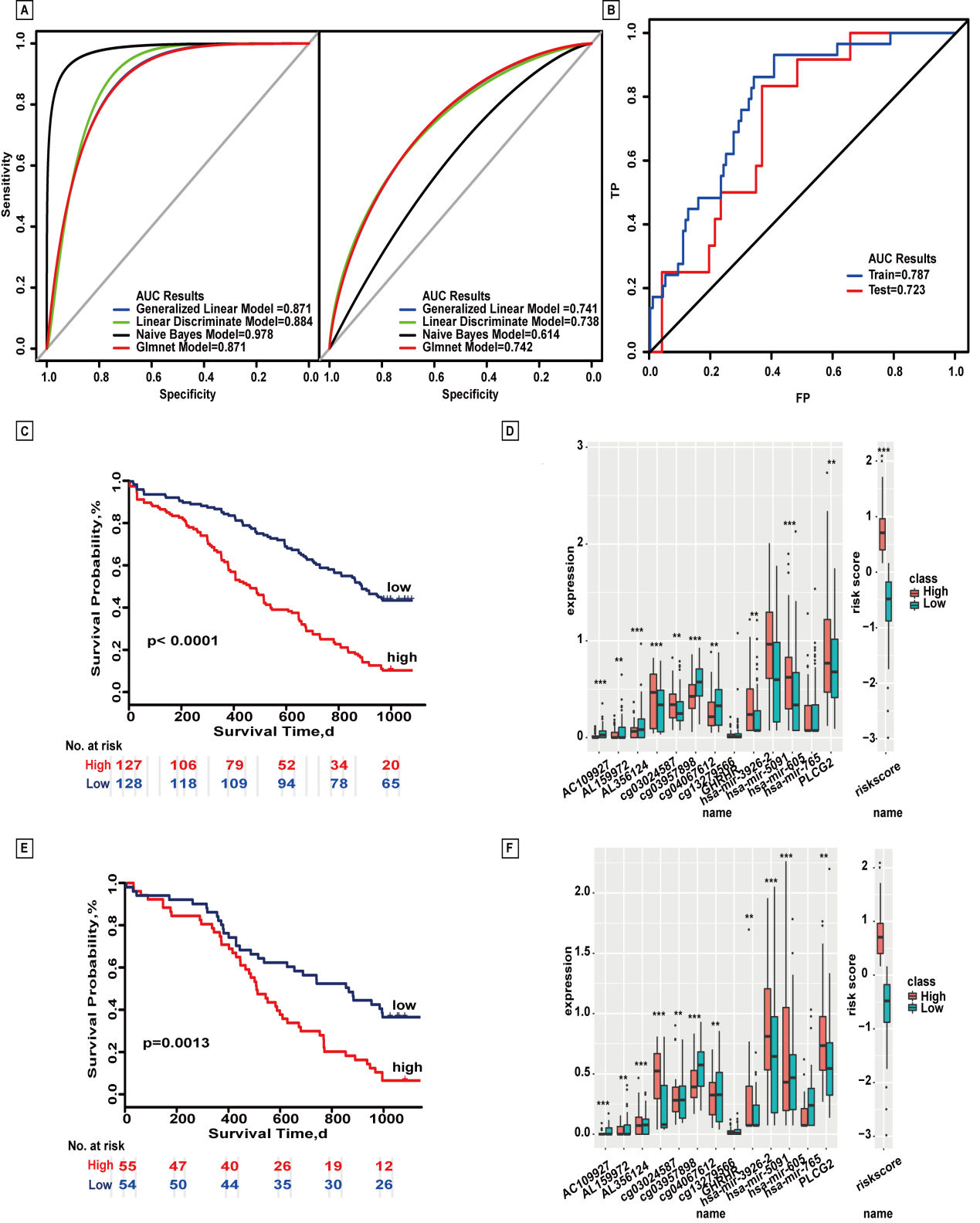


Supplementary Figure17. Important molecular features for 3-year survival based on TCGA database. (A) ROC curves of the training set and test set. (B) ROC survival curves of the two sets. (C,E) Kaplan-Meier survival curves of the two sets. (D,F) Distribution of molecular expression in high- and low-risk groups of the two sets. ***means p<0.01,**means p<0.05.


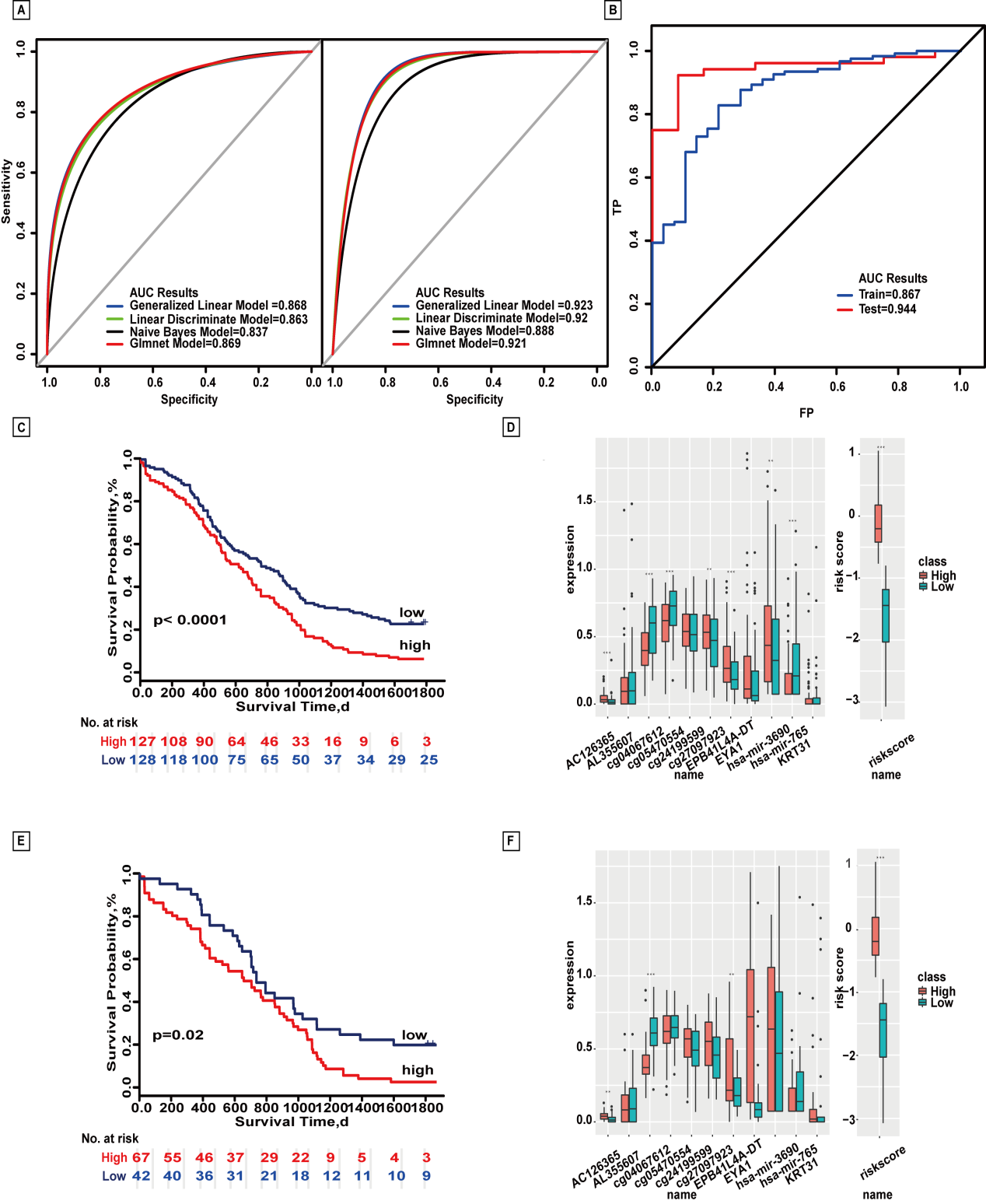


Supplementary Figure18. Important molecular features for 5-year survival based on TCGA database. (A) ROC curves of the training set and test set. (B) ROC survival curves of the two sets. (C,E) Kaplan-Meier survival curves of the two sets. (D,F) Distribution of molecular expression in high- and low-risk groups of the two sets. ***means p<0.01,**means p<0.05.


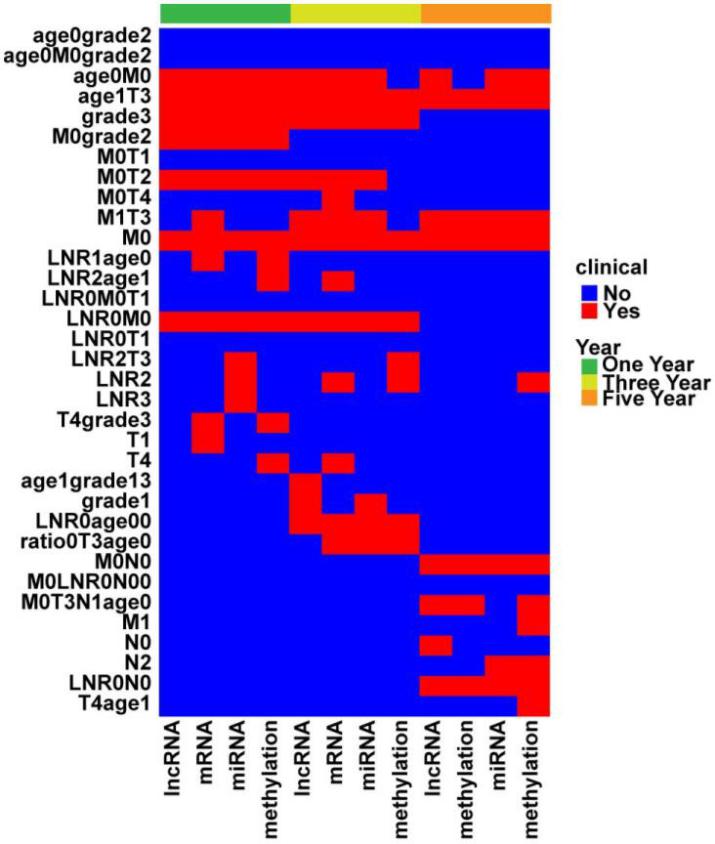


Supplementary Figure19.Screening combinations of molecular features and clinical characteristics for survival time.The rows of heatmap were shown combinations clinical characteristics for1-year ,3-year and 5-year survival, the columns of heatmap were shown molecular types and survival time. Molecules divided combinations of clinical characteristics according to the survival time, the color of the heat map is red during this survival time, and the rest were blue.


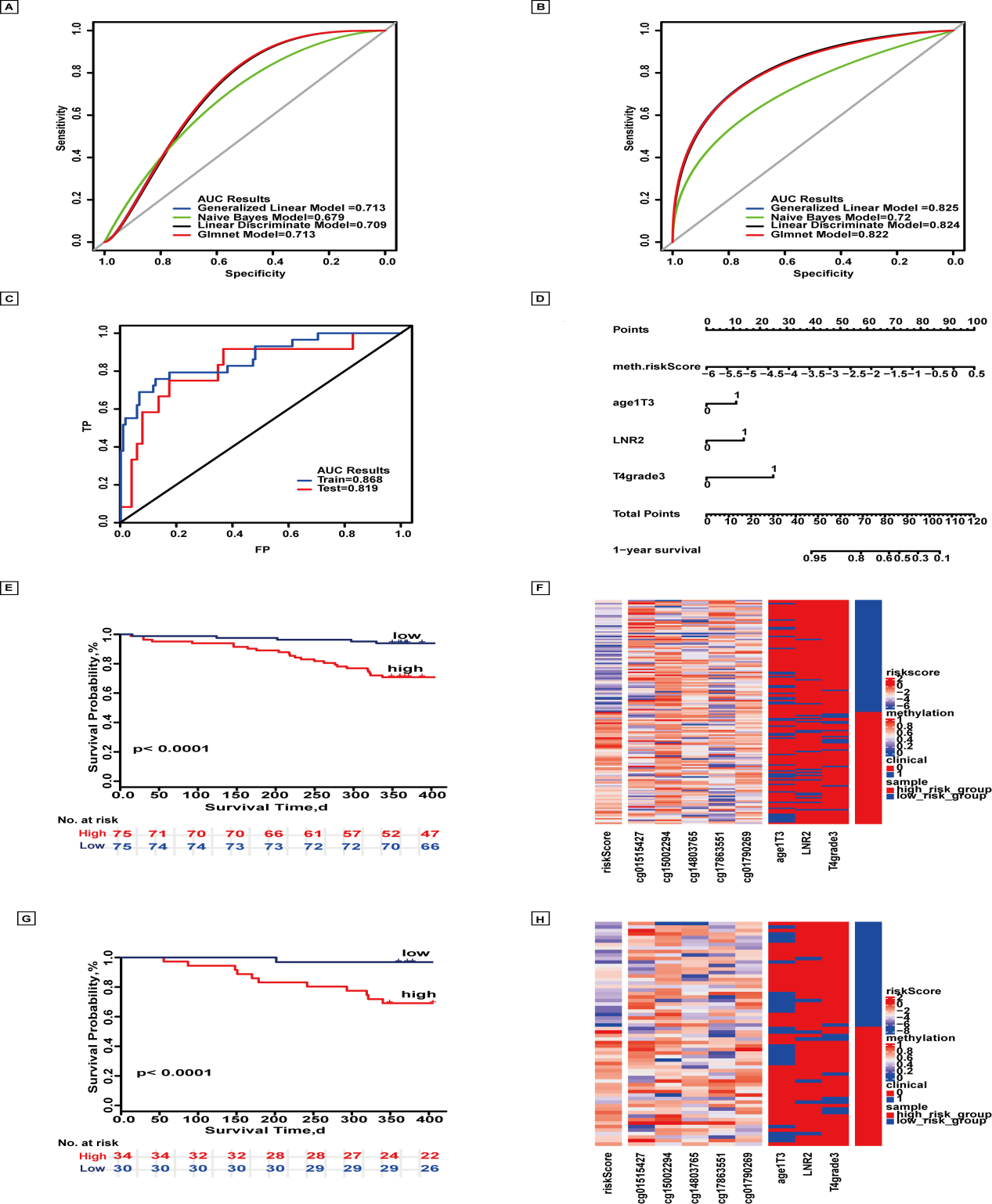


Supplementary Figure20. Important combinations of clinical characteristics and DNA methylation site based on TCGA database for 1-year survival time. (A,B) ROC curves of the training set and test set.(C) ROC survival curves of the two sets. (D) Nomogram of multivariate Cox regression. (E,G) Kaplan-Meier survival curves of the two sets.(F,H) Heatmaps of the two sets used to compare the differences between high- and low-risk groups.


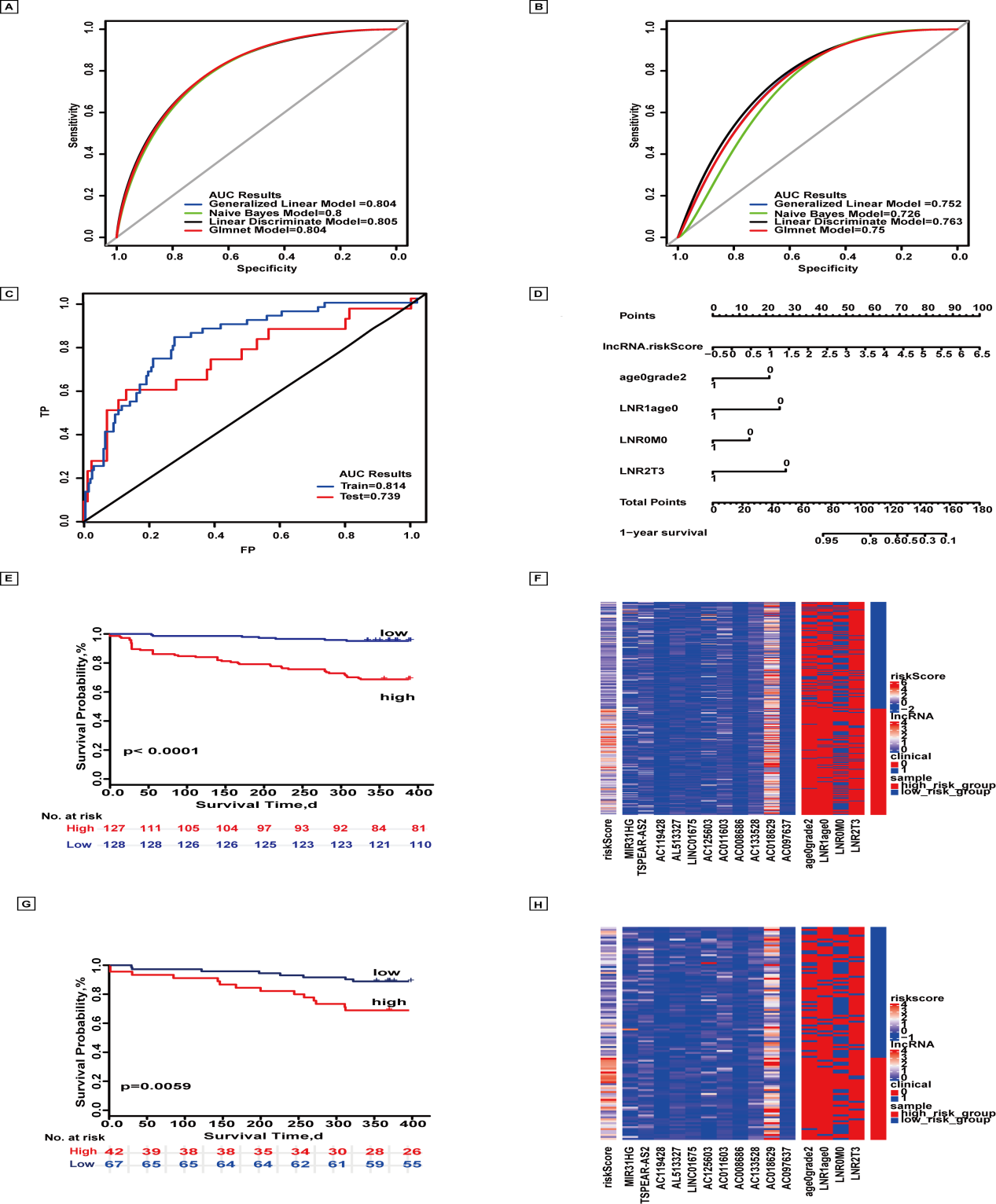


Supplementary Figure21. Important combinations of clinical characteristics and lncRNA based on TCGA database for 1-year survival time.(A,B) ROC curves of the training set and test set. (C) ROC survival curves of the two sets. (D) Nomogram of multivariate Cox regression. (E,G) Kaplan-Meier survival curves of the two sets.(F,H) Heatmaps of the two sets used to compare the differences between high- and low-risk groups
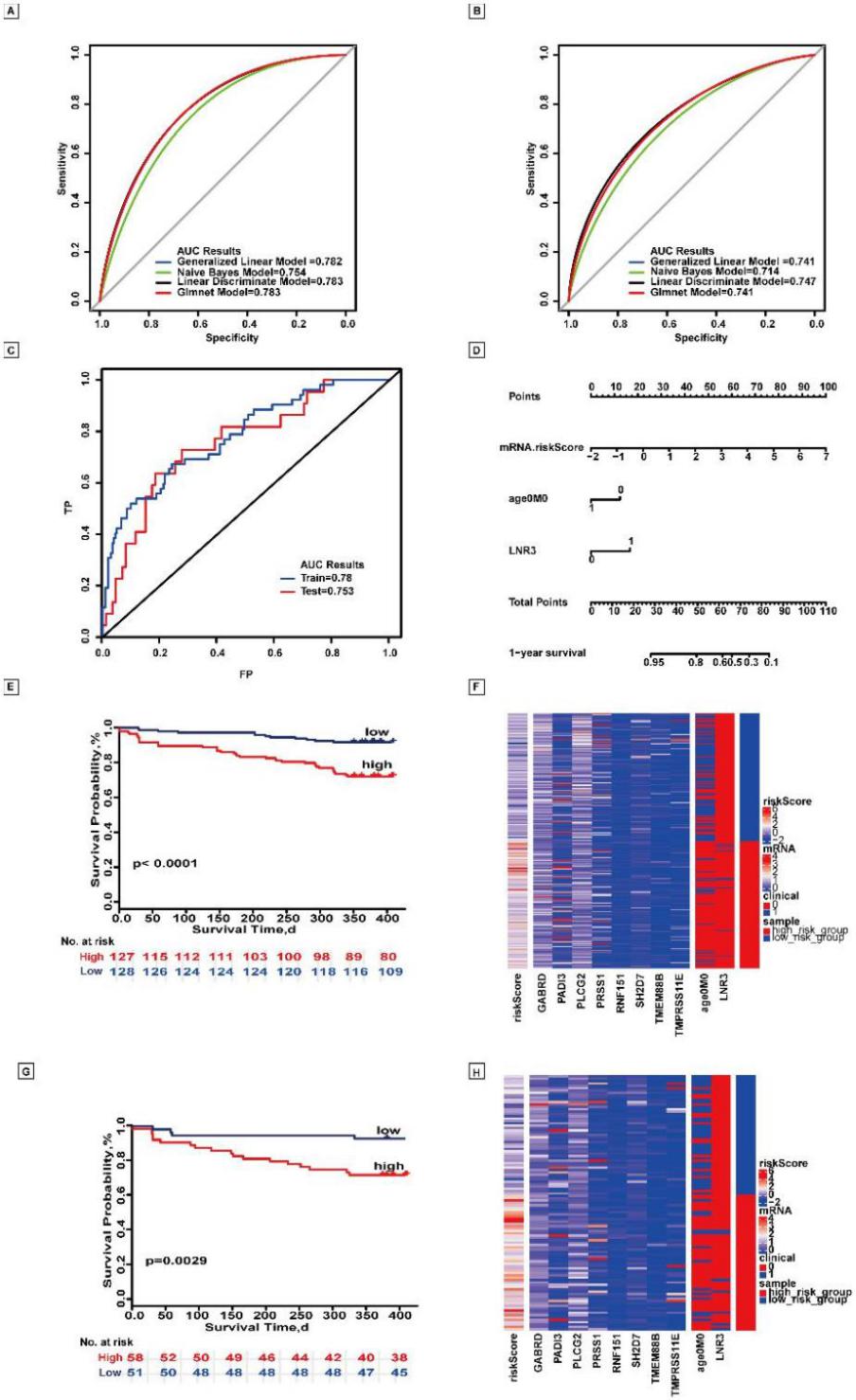
.


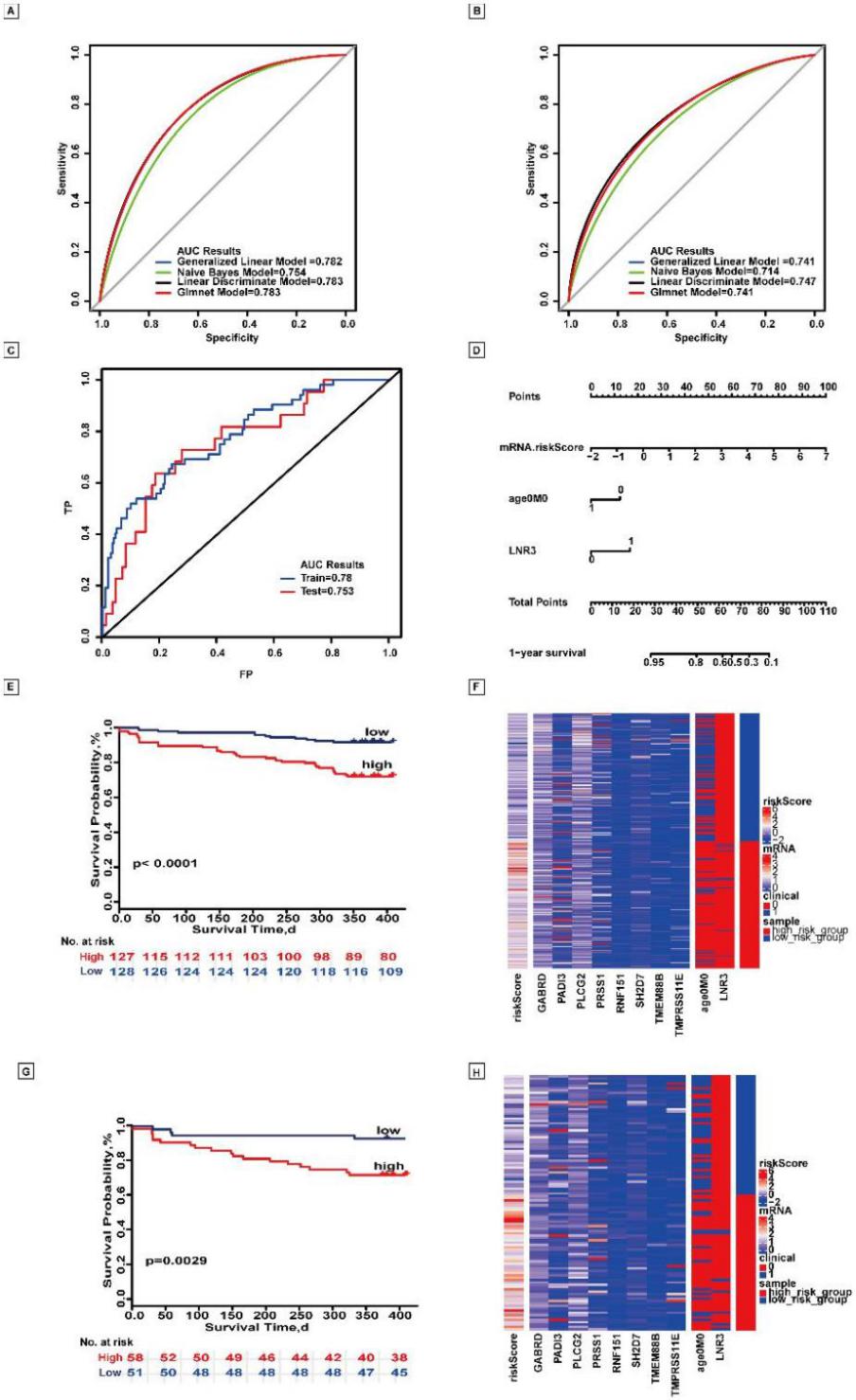


Supplementary Figure22. Important combinations of clinical characteristics and mRNA based on TCGA database for 1-year survival time.(A,B) ROC curves of the training set and test set.(C)ROC survival curves of the two sets.(D) Nomogram of multivariateCox regression. (E,G) Kaplan-Meier survival curves of the two sets.(F,H) Heatmaps of the two sets used to compare the differences between high- and low-risk groups .


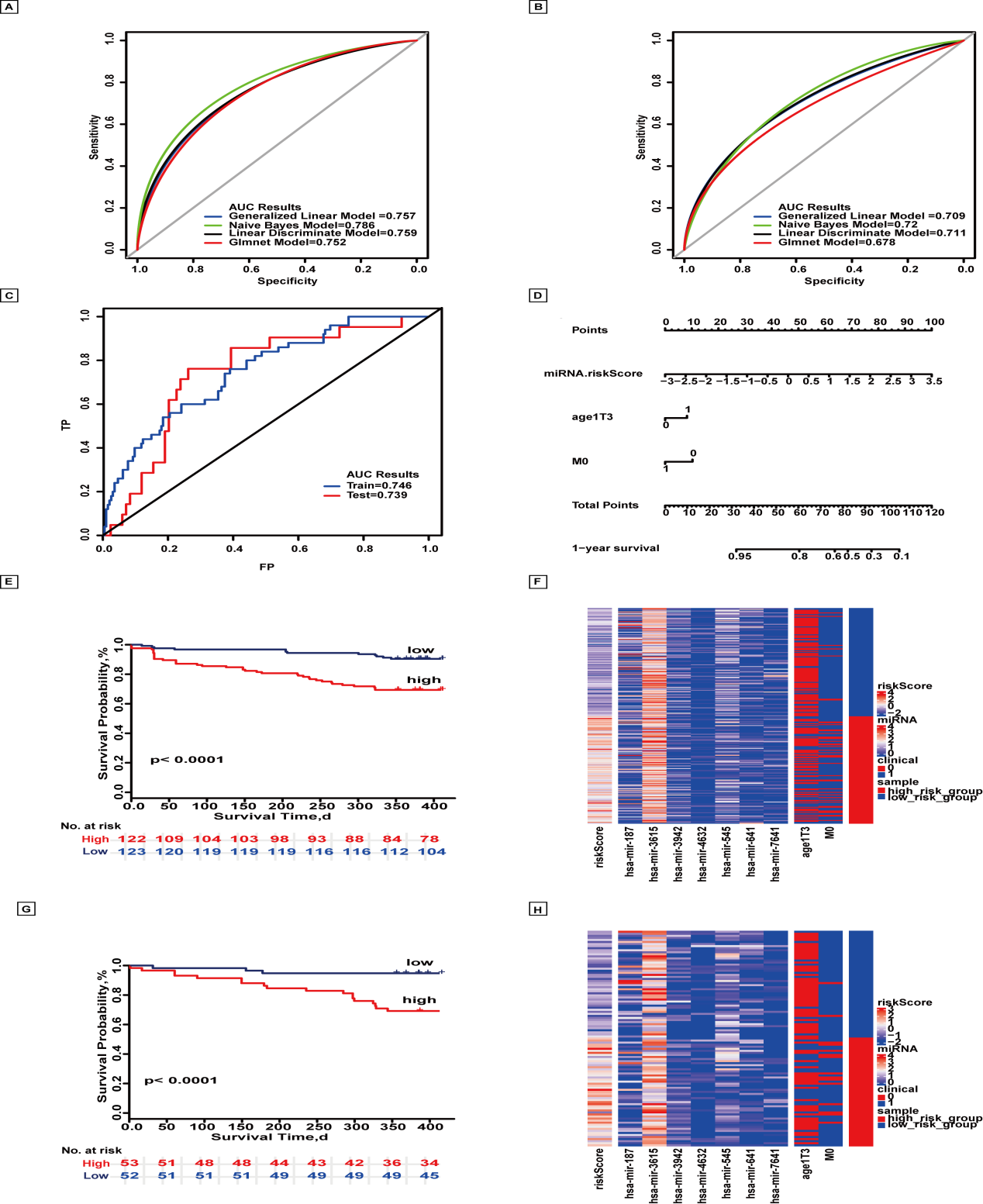
Supplementary Figure23. Important combinations of clinical characteristics and miRNA based on TCGA database for 1-year survival time. (A,B) ROC curves of the training set and test set. (C) ROC survival curves of the two sets. (D) Nomogram of multivariate Cox regression. (E,G) Kaplan-Meier survival curves of the two sets.(F,H) Heatmaps of the two sets used to compare the differences between high- and low-risk groups.
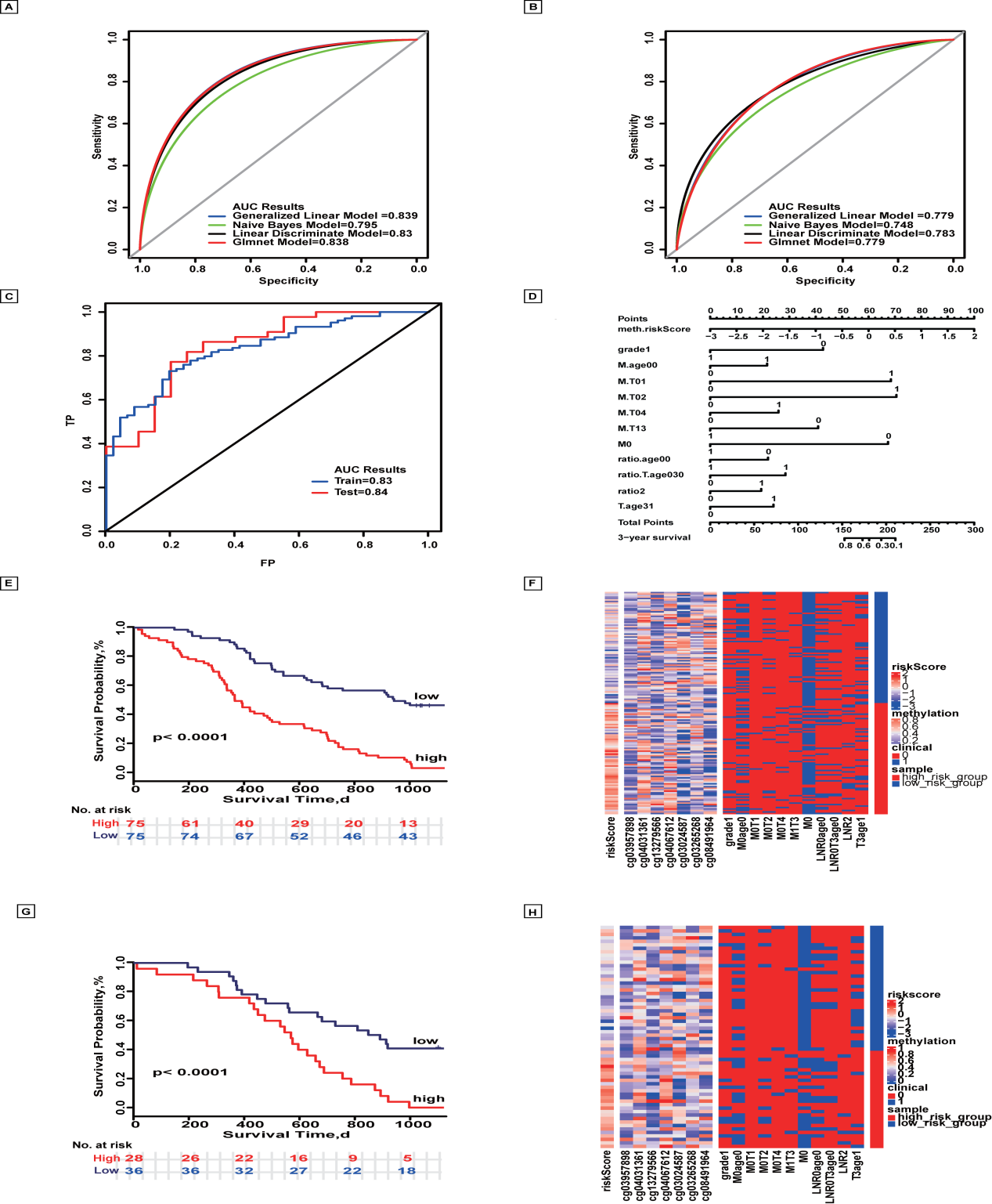


Supplementary Figure24. Important combinations of clinical characteristics and DNA methylation site based on TCGA database for 3-year survival time. (A,B) ROC curves of the training set and test set. (C) ROC survival curves of the two sets. (D) Nomogram of multivariate Cox regression. (E,G) Kaplan-Meier survival curves of the two sets. (F,H) Heatmaps of the two sets used to compare the differences between high- and low-risk groups.


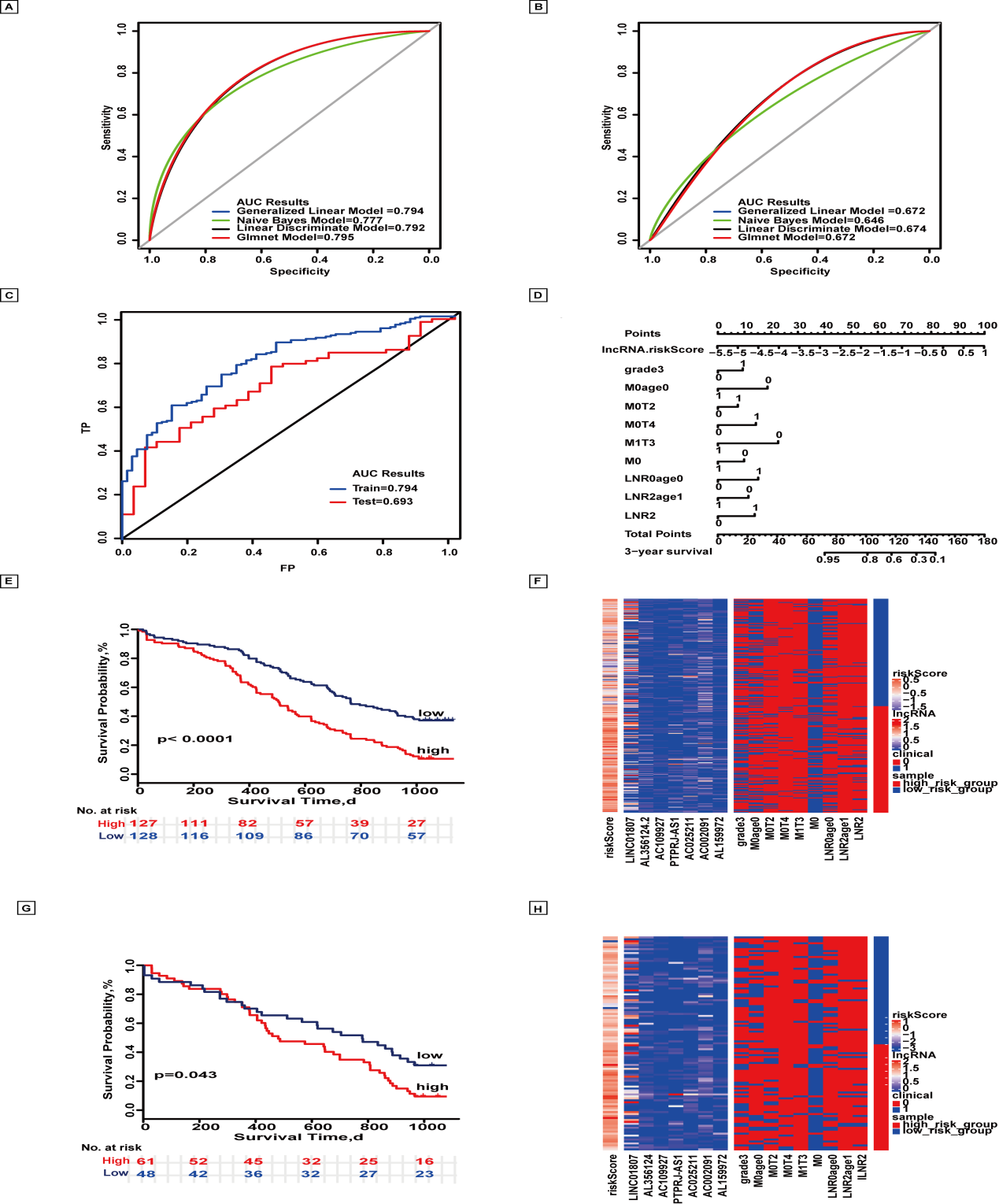


Supplementary Figure25. Important combinations of clinical characteristics and lncRNA based on TCGA database for 3-year survival time. (A,B) ROC curves of the training set and test set. (C) ROC survival curves of the two sets. (D) Nomogram of multivariate Cox regression. (E,G) Kaplan-Meier survival curves of the two sets. (F,H) Heatmaps of the two sets used to compare the differences between high- and low-risk groups.


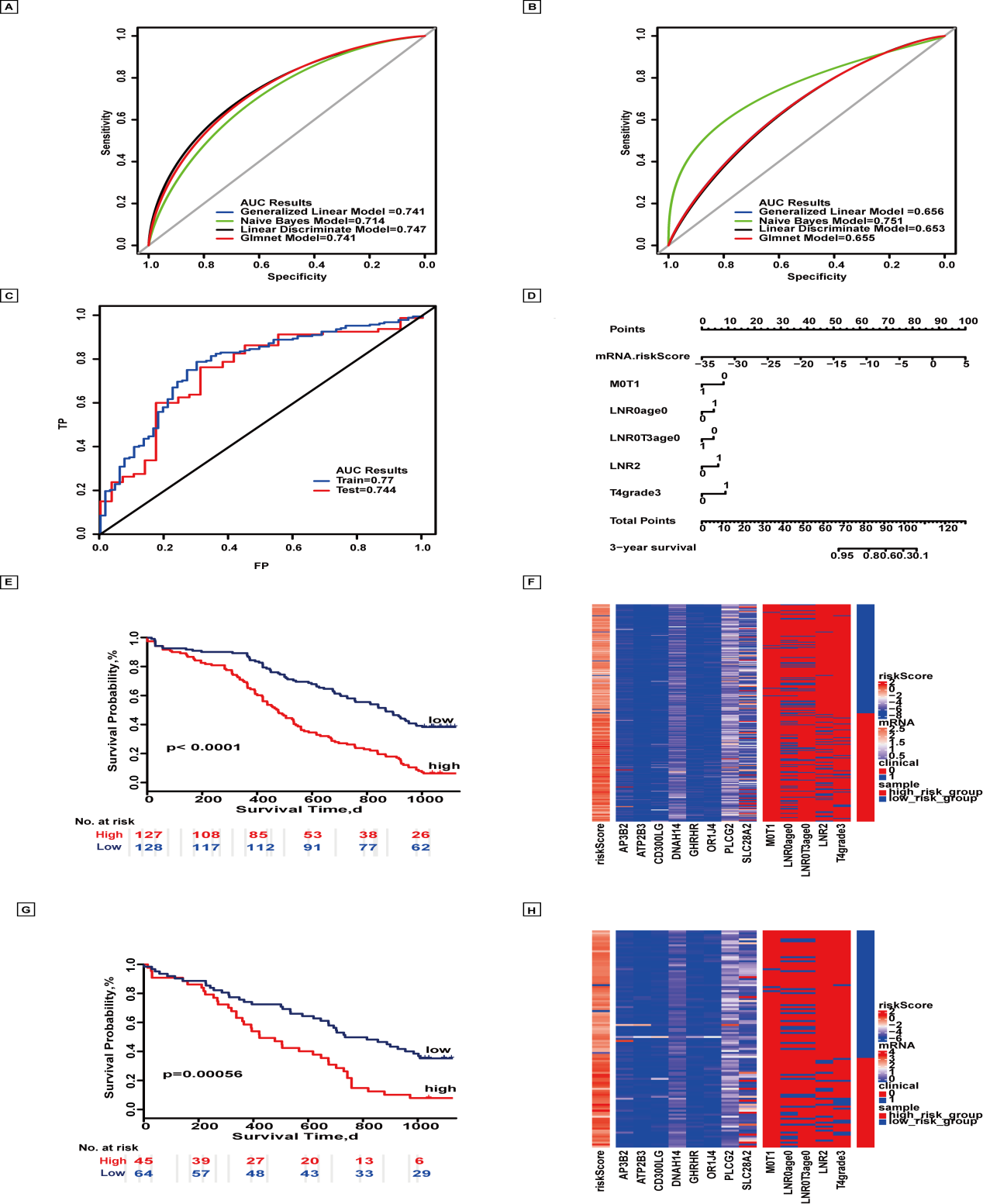


Supplementary Figure26. Important combinations of clinical characteristics and mRNA based on TCGA database for 3-year survival time . (A,B)ROC curves of the training set and test set. (C) ROC survival curves of the two sets. (D) Nomogram of multivariate Cox regression. (E,G) Kaplan-Meier survival curves of the two sets. (F,H) Heatmaps of the two sets used to compare the differences between high- and low-risk groups.


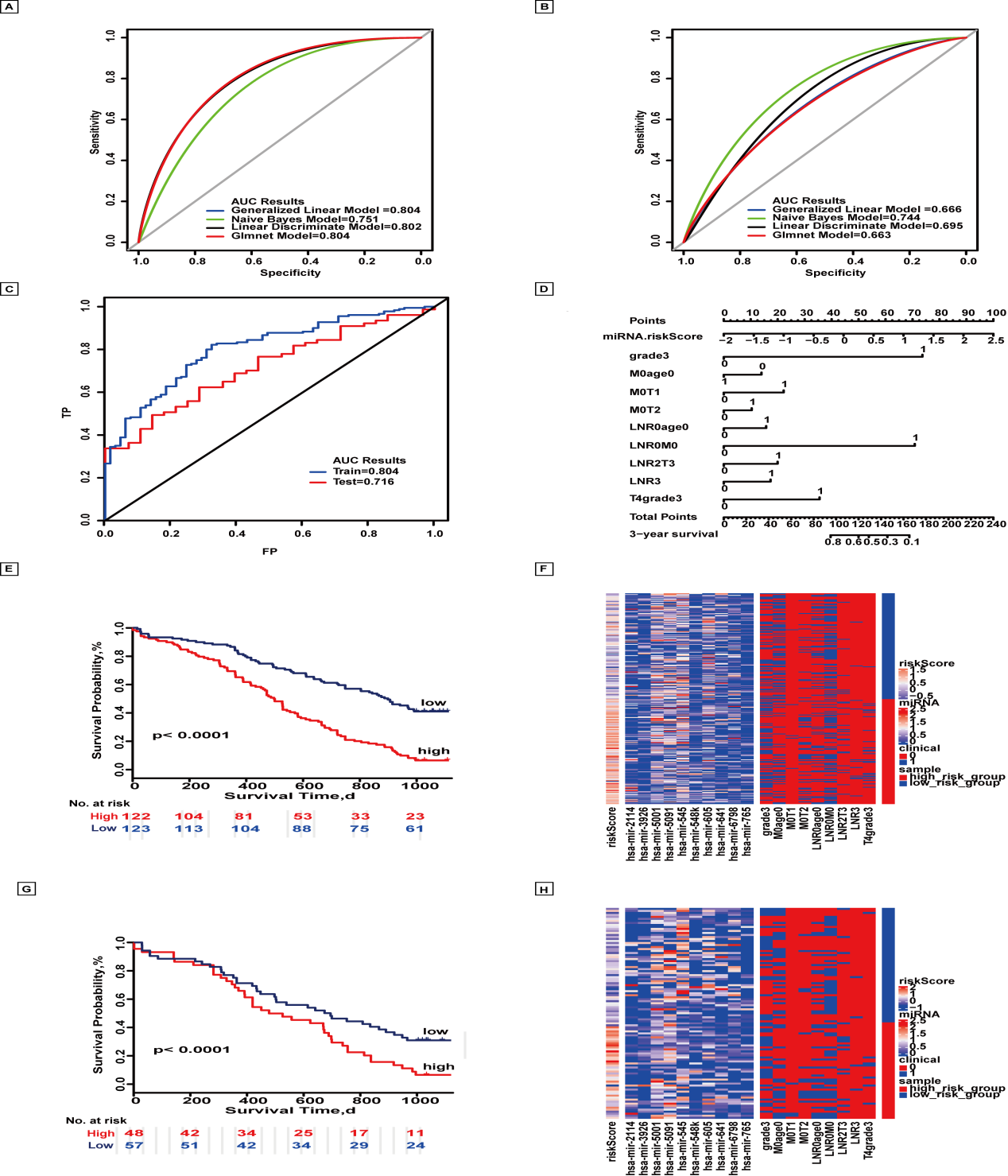


Supplementary Figure27. Important combinations of clinical characteristics and miRNA based on TCGA database for 3-year survival time.(A,B)ROC curves of the training set and test set.(C) ROC survival curves of the two sets.(D) Nomogram of multivariate Cox regression. (E,G) Kaplan-Meier survival curves of the two sets. (F,H) Heatmaps of the two sets used to compare the differences between high- and low-risk groups.


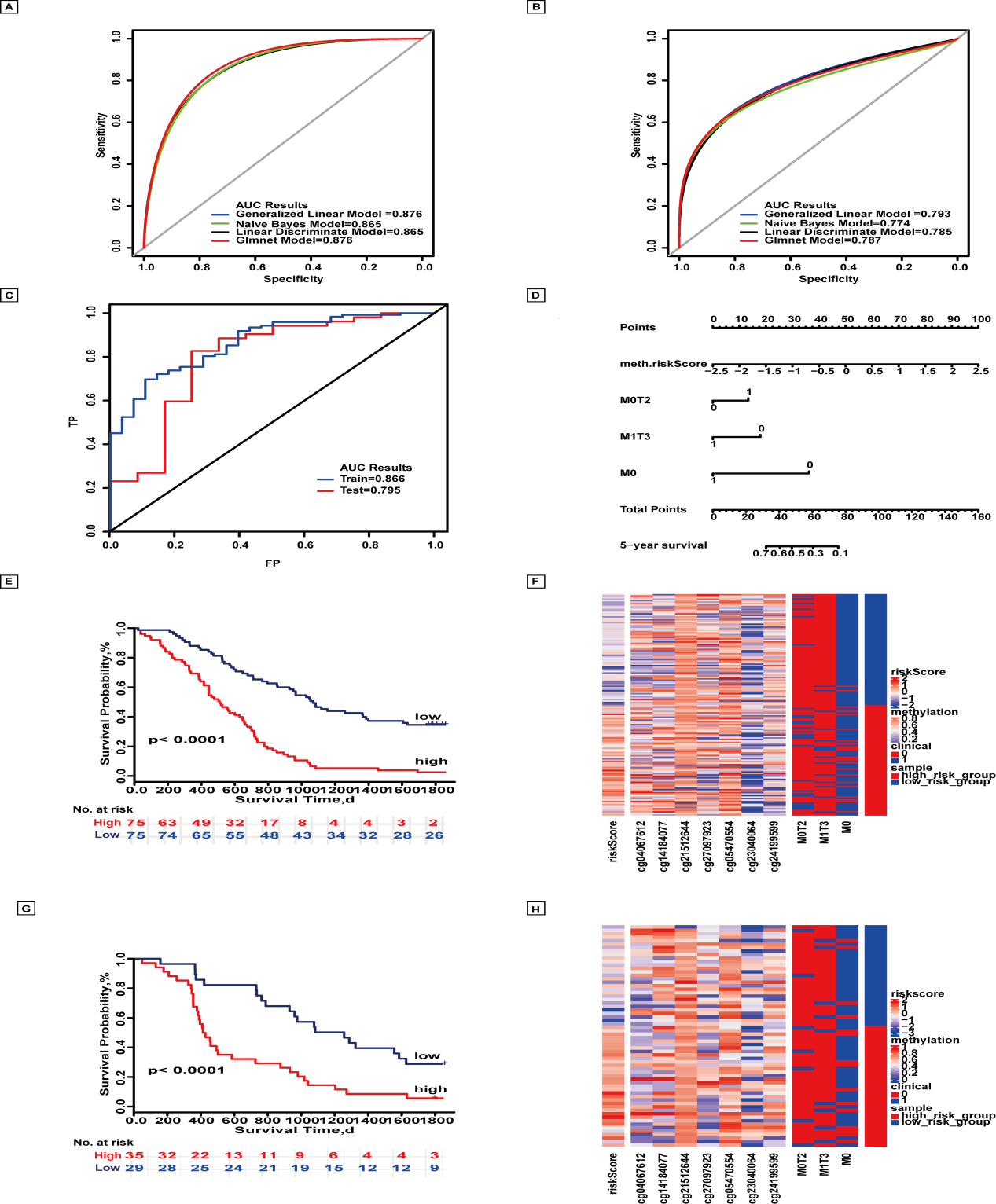


Supplementary Figure28. Important combinations of clinical characteristics and DNA methylation site based on TCGA database for 5-year survival time . (A,B) ROC curves of the training set and test set. (C) ROC survival curves of the two sets. (D) Nomogram of multivariate Cox regression.(E,G) Kaplan-Meier survival curves of the two sets. (F,H) Heatmaps of the two sets used to compare the differences between high- and low-risk groups.


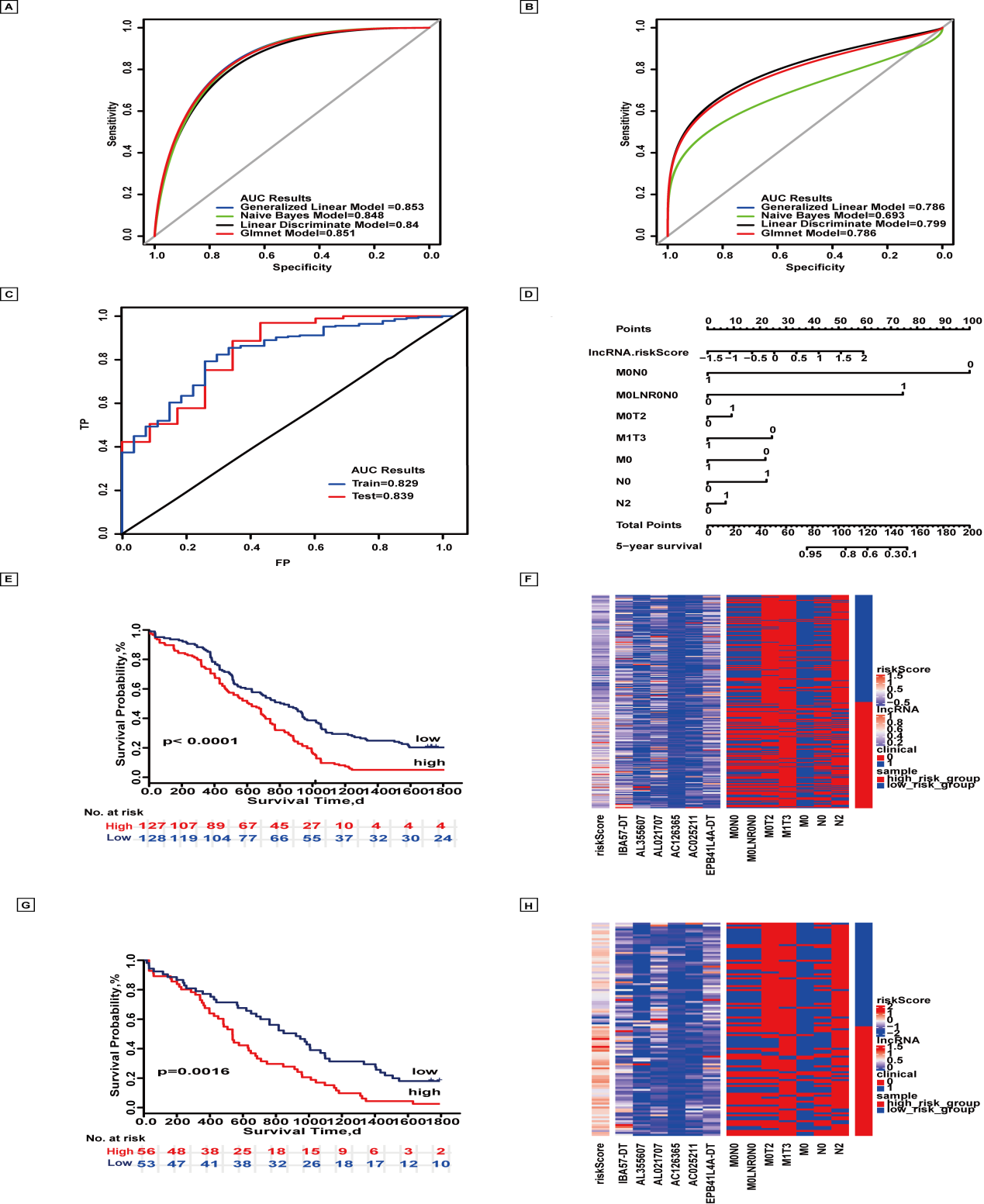


Supplementary Figure29. Important combinations of clinical characteristics and lncRNA based on TCGA database for 5-year survival time.(A,B) ROC curves of the training set and test set.(C) ROC survival curves of the two sets.(D) Nomogram of multivariate Cox regression. (E,G) Kaplan-Meier survival curves of the two sets.(F,H) Heatmaps of the two sets used to compare the differences between high- and low-risk groups.


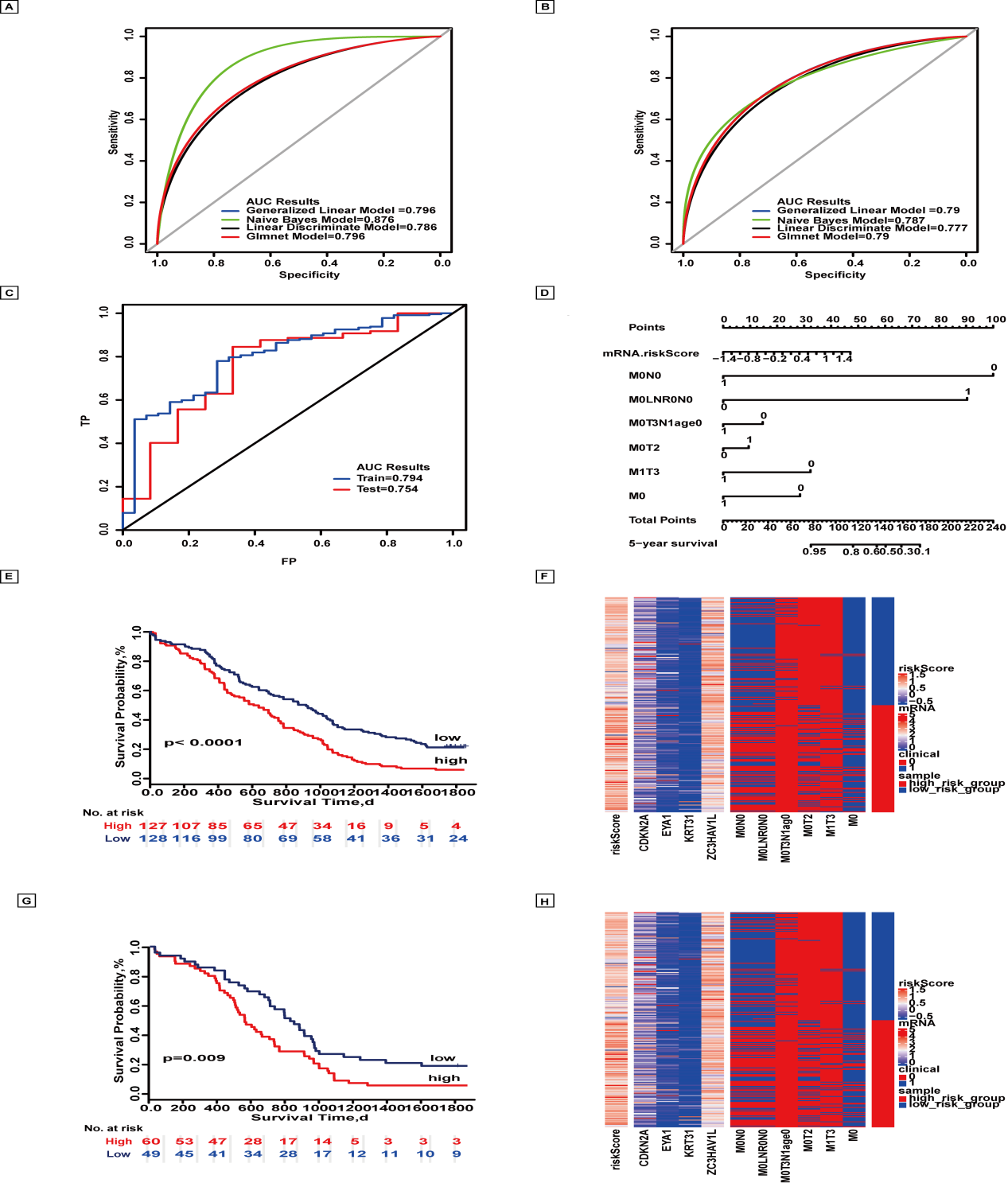


Supplementary Figure30. Important combinations of clinical characteristics and mRNA based on TCGA database for 5-year survival time. (A,B) ROC curves of the training set and test set.(C) ROC survival curves of the two sets. (D) Nomogram of multivariate Cox regression. (E,G) Kaplan-Meier survival curves of the two sets.(F,H) Heatmaps of the two sets used to compare the differences between high- and low-risk groups.


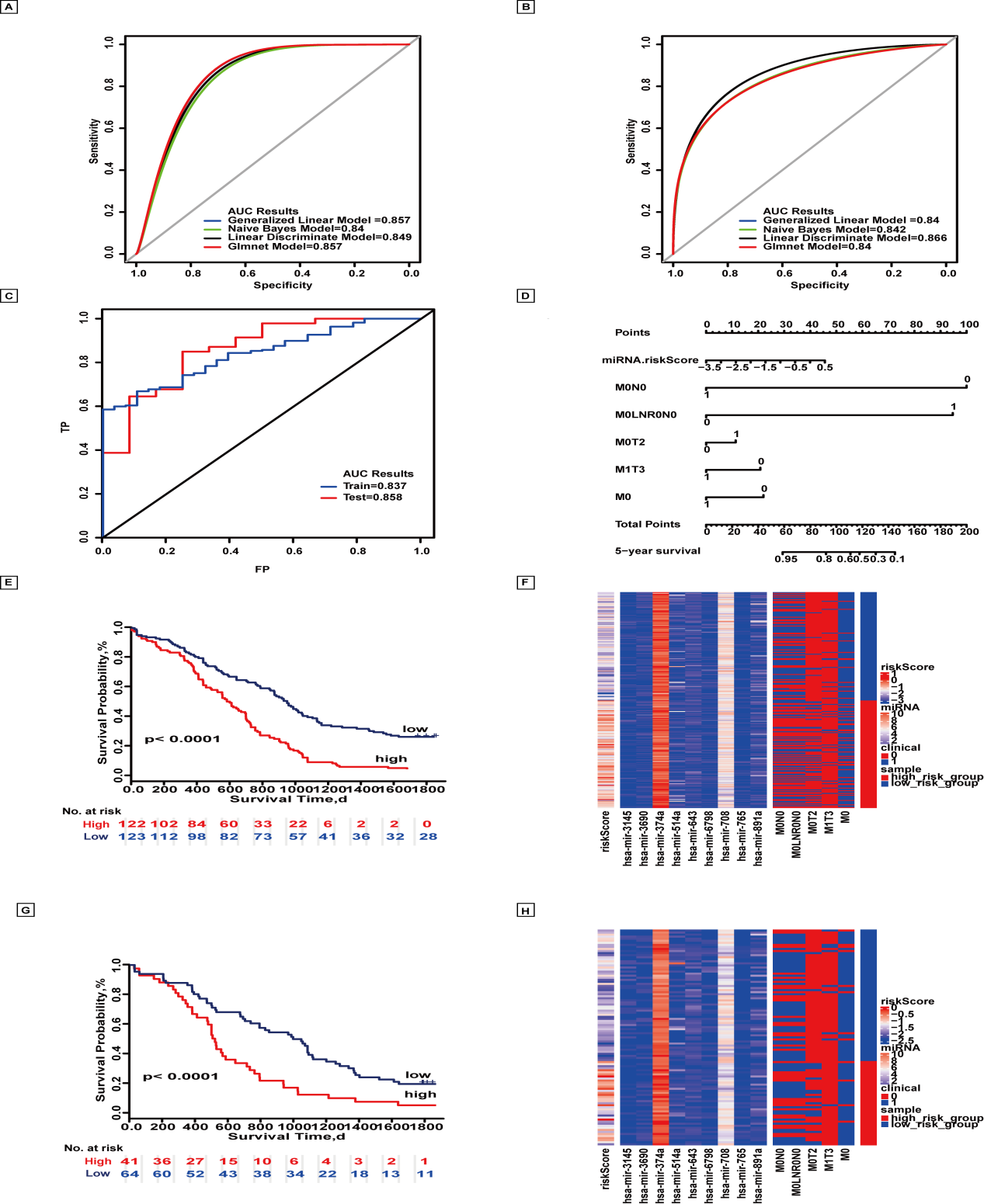


Supplementary Figure31. Important combinations of clinical characteristics and miRNA based on TCGA database for 5-year survival time. (A,B) ROC curves of the training set and test set. (C) ROC survival curves of the two sets.(D) Nomogram of multivariate Cox regression. (E,G) Kaplan-Meier survival curves of the two sets. (F,H) Heatmaps of the two sets used to compare the differences between high- and low-risk groups.
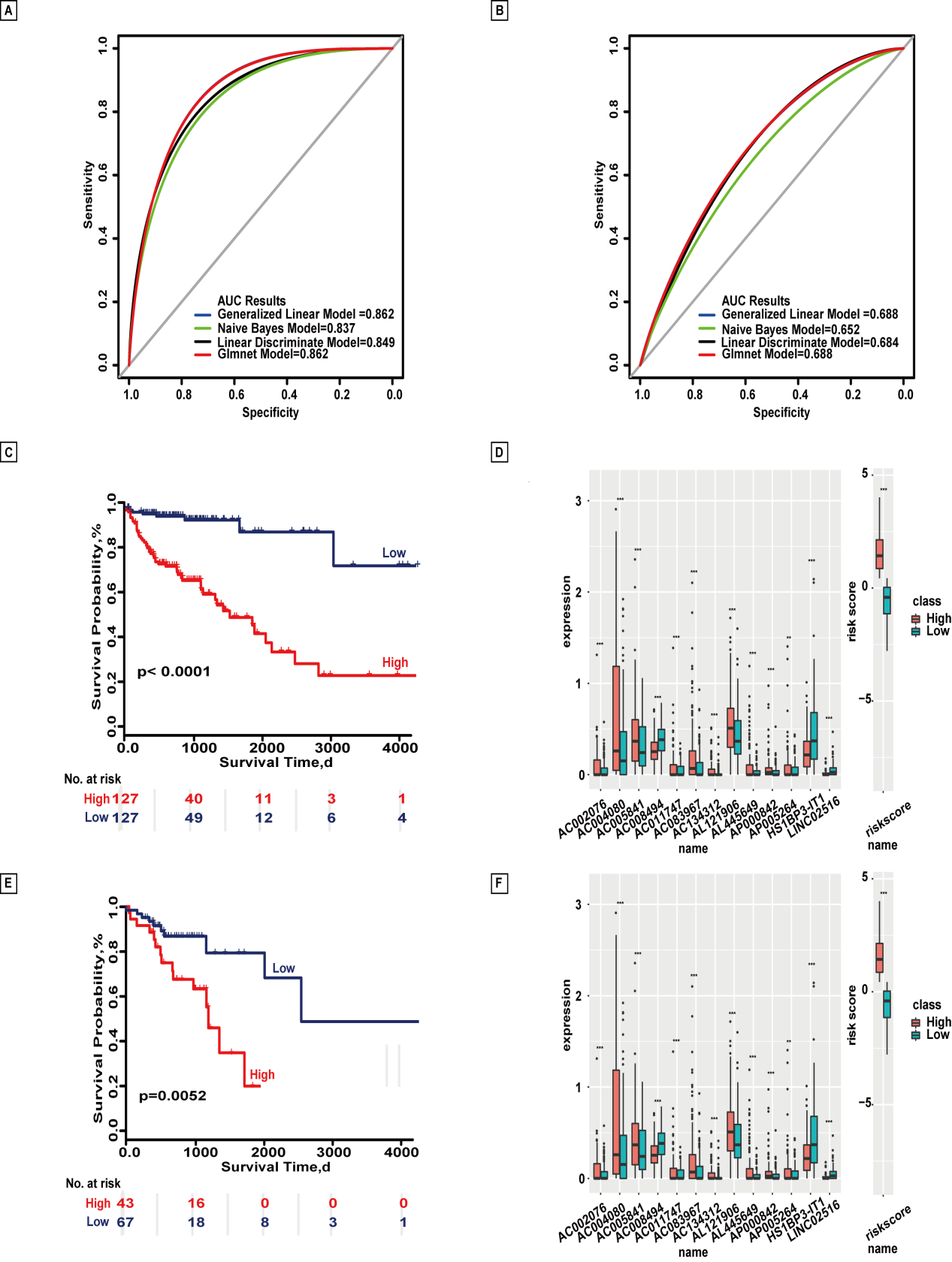


Supplementary Figure32.Important lncRNA features for overall survival based on TCGA database. (A,B) ROC curves of the training set and test set. (C,E) Kaplan-Meier survival curves of the two sets. (D,F) Distribution of lncRNA expression in high- and low-risk groups of the two sets.***means p<0.01, **means p<0.05.


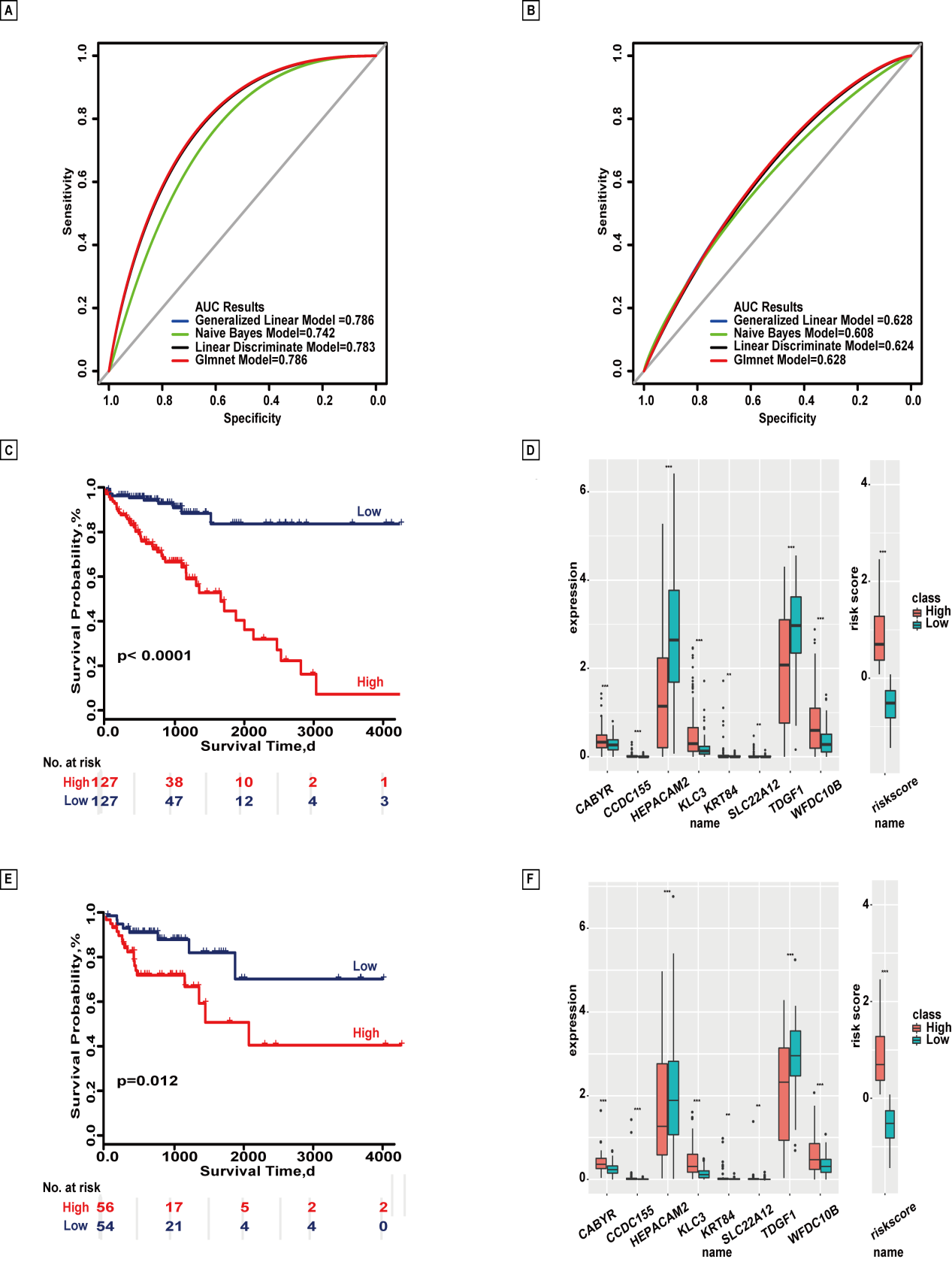


Supplementary Figure33. Important mRNA features for overall survival based on TCGA database. (A,B) ROC curves of the training set and test set. (C,E) Kaplan-Meier survival curves of the two sets. (D,F) Distribution of lncRNA expression in high- and low-risk groups of the two sets.***means p<0.01, **means p<0.05.


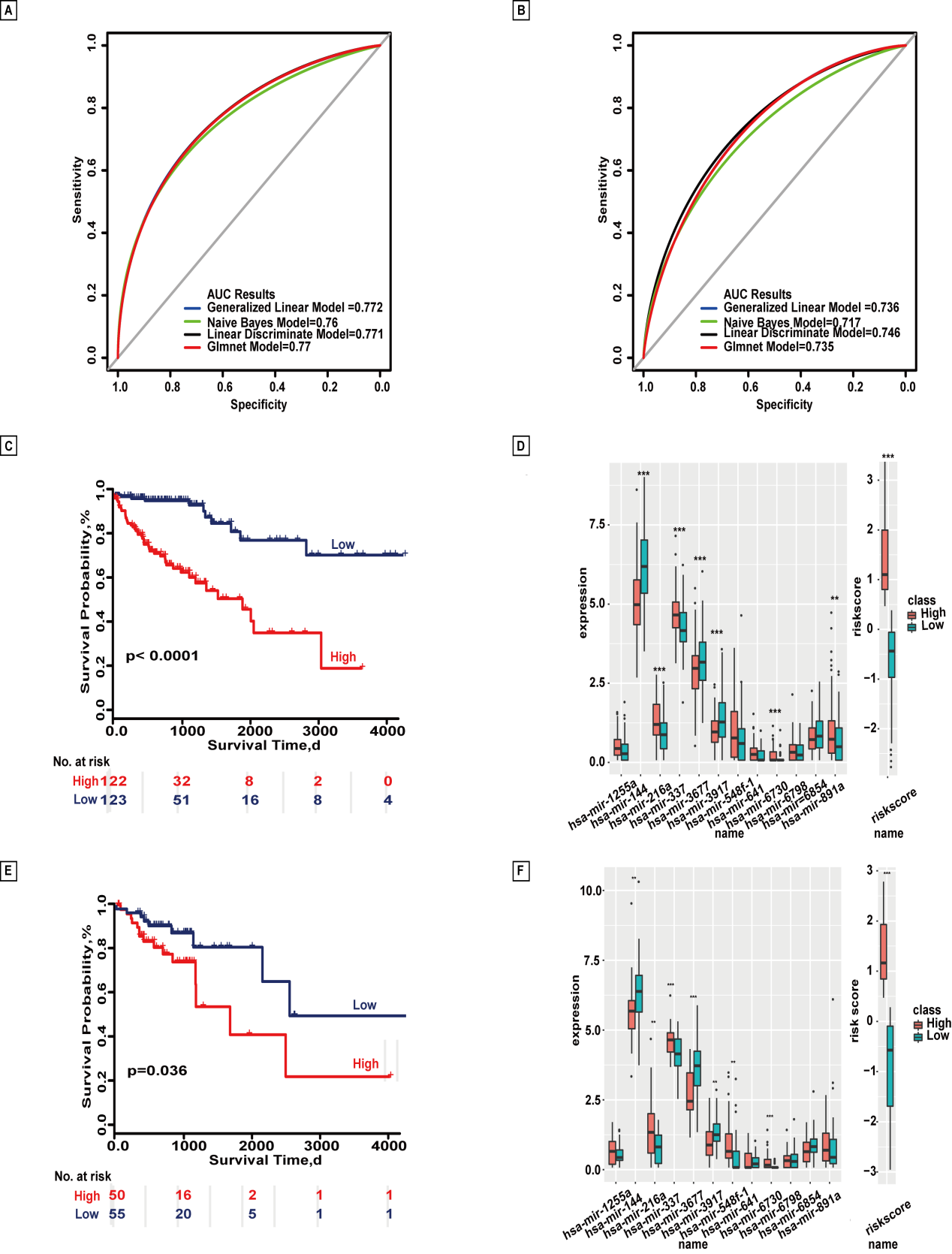


Supplementary Figure34. Important miRNA features for overall survival based on TCGA database. (A,B) ROC curves of the training set and test set. (C,E) Kaplan-Meier survival curves of the two sets. (D,F) Distribution of lncRNA expression in high- and low-risk groups of the two sets.***means p<0.01, **means p<0.05.


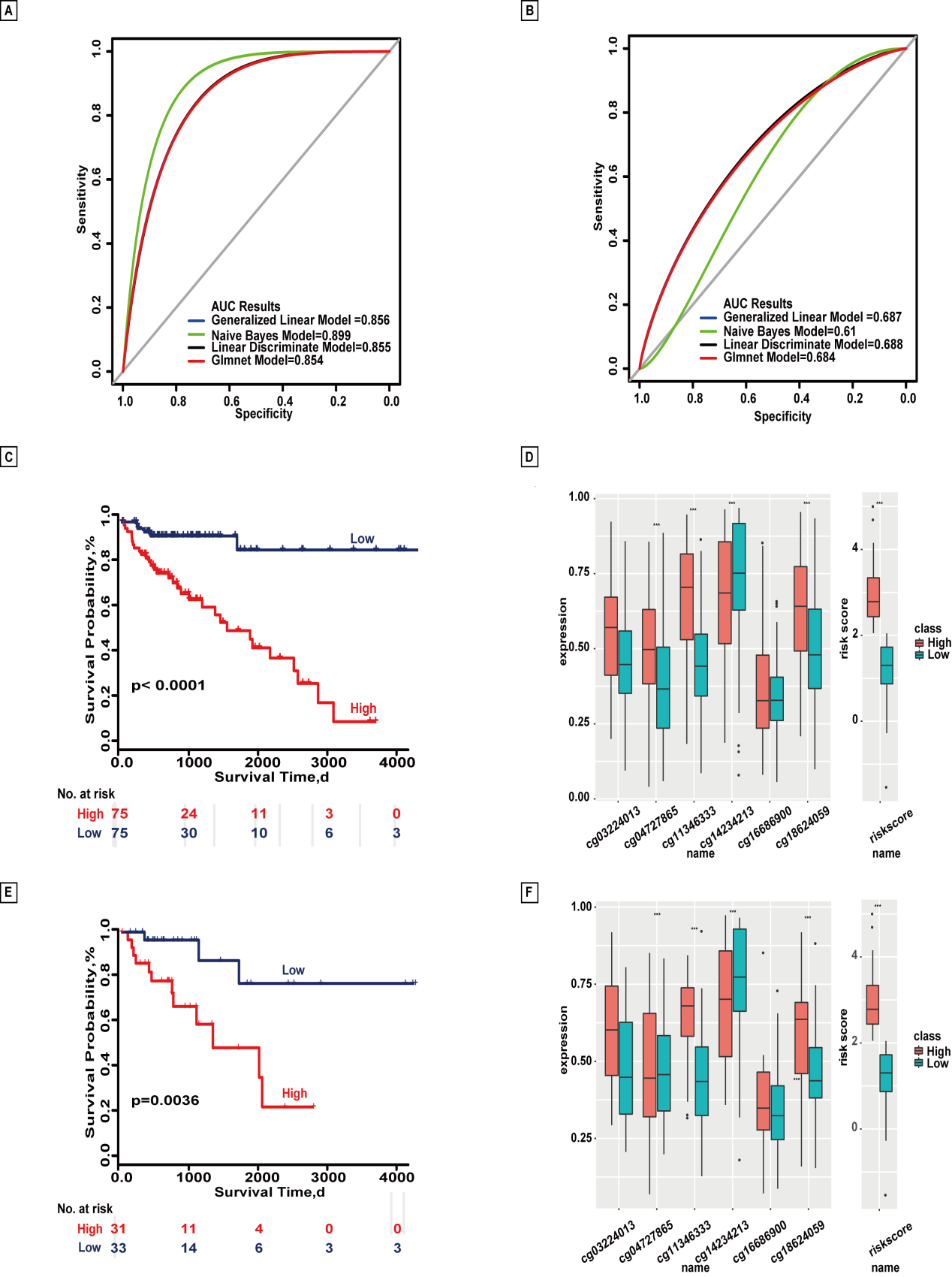


Supplementary Figure35. Important DNA methylation features for overall survival based on TCGA database. (A,B) ROC curves of the training set and test set. (C,E) Kaplan-Meier survival curves of the two sets. (D,F) Distribution of lncRNA expression in high- and low-risk groups of the two sets.***means p<0.01, **means p<0.05.


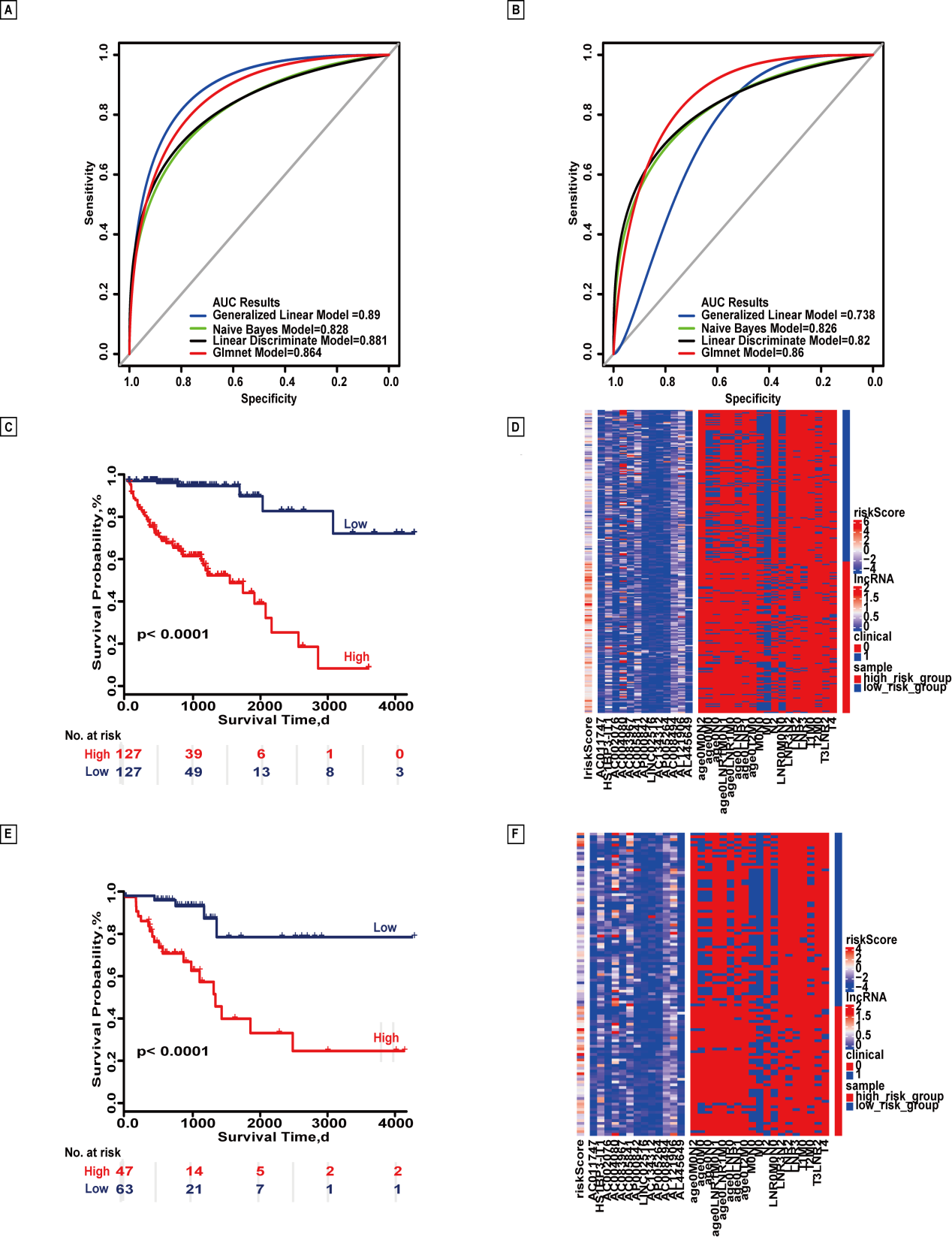


Supplementary Figure36. Important combinations of clinical characteristics and lncRNA for overall survival based on TCGA database. (A,B) ROC curves of the training set and test set. (C,E) Kaplan-Meier survival curves of the two sets. (D,F) Heatmaps of the two sets used to compare the differences between high- and low-risk groups.


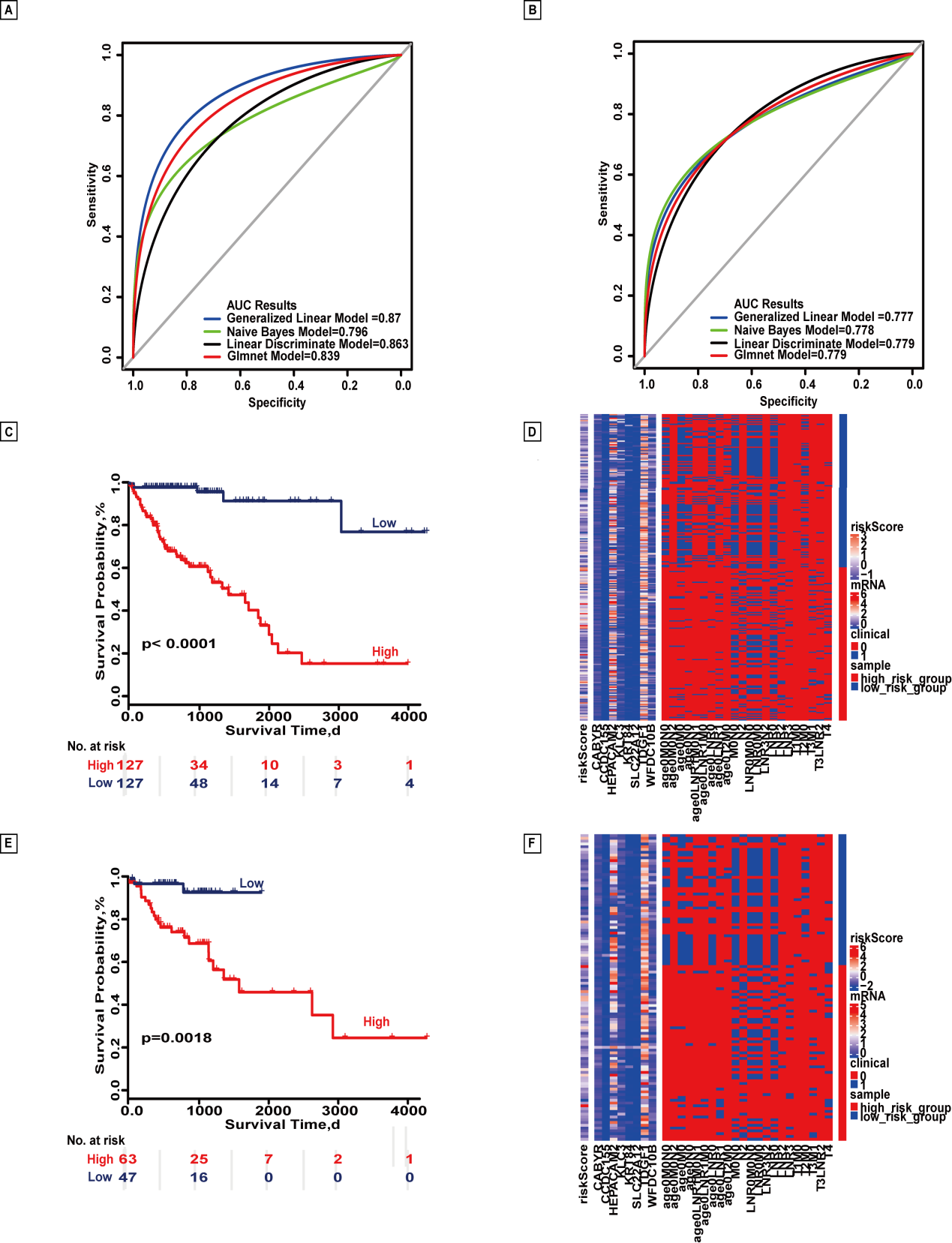


Supplementary Figure37. Important combinations of clinical characteristics and mRNA for overall survival based on TCGA database.(A,B) ROC curves of the training set and test set. (C,E) Kaplan-Meier survival curves of the two sets. (D,F) Heatmaps of the two sets used to compare the differences between high- and low-risk groups.
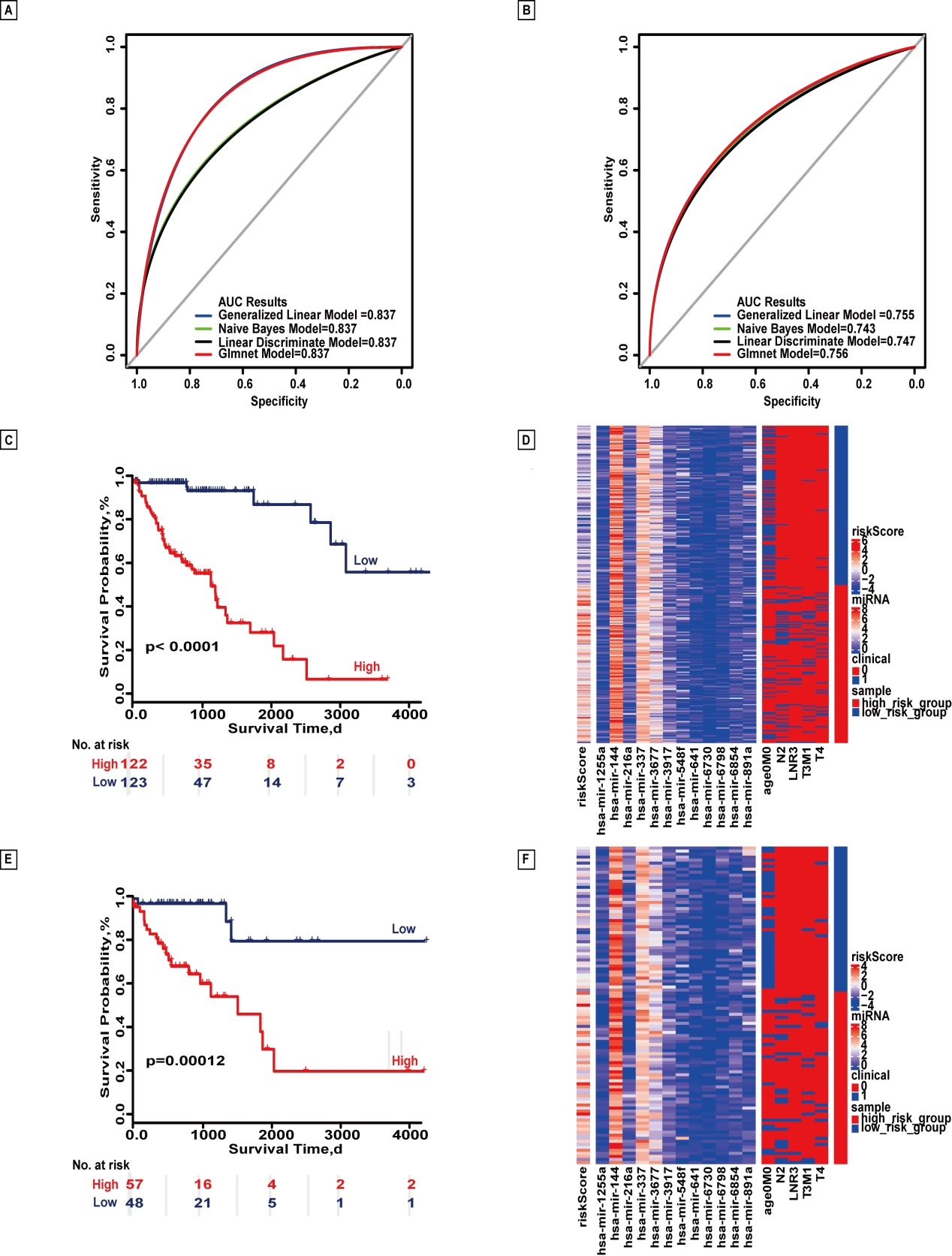


Supplementary Figure38. Important combinations of clinical characteristics and miRNA for overall survival based on TCGA database. (A,B) ROC curves of the training set and test set. (C,E) Kaplan-Meier survival curves of the two sets. (D,F) Heatmaps of the two sets used to compare the differences between high- and low-risk groups.


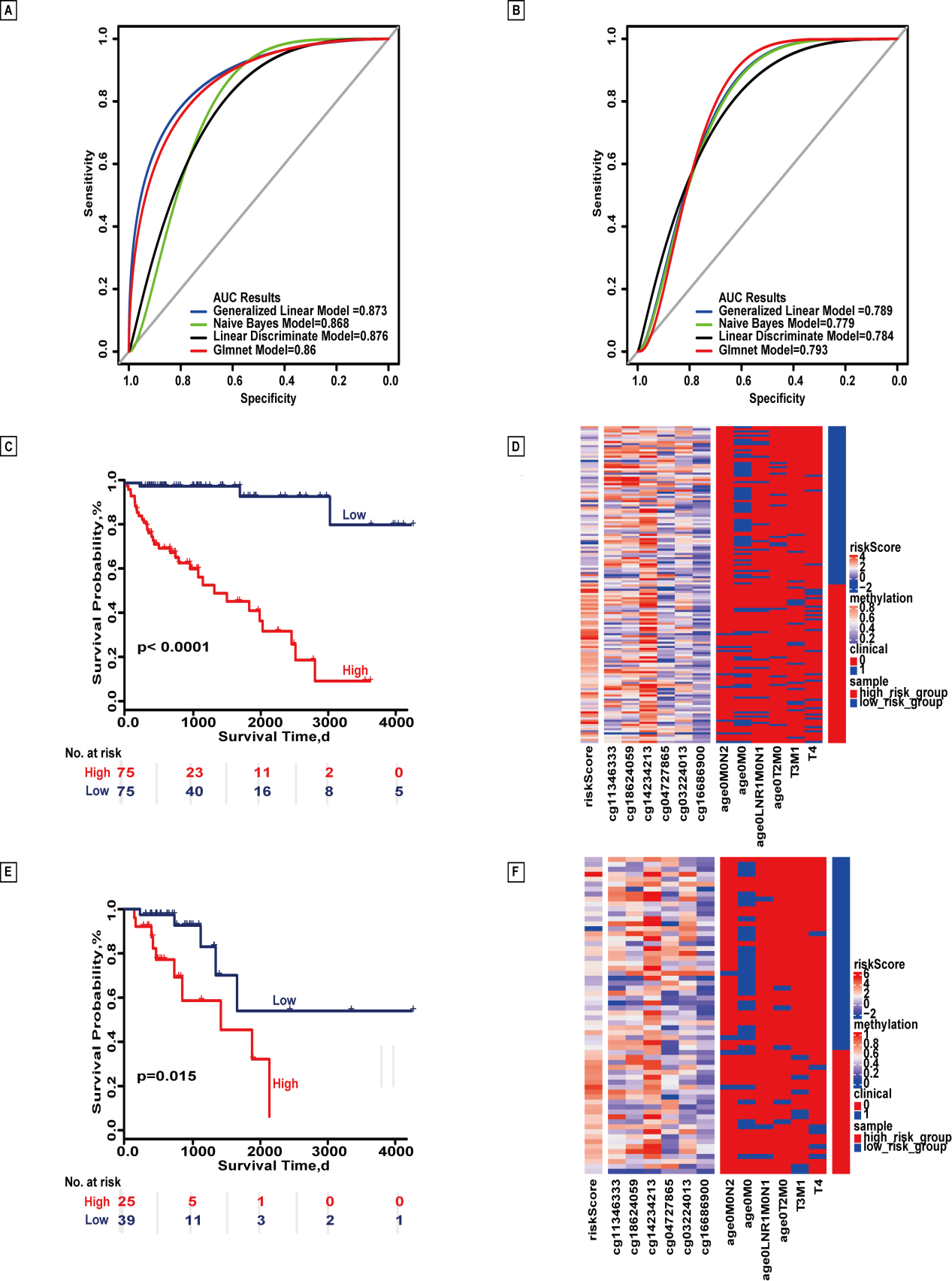


Supplementary Figure39. Important combinations of clinical characteristics and DNA methylation site for overall survival based on TCGA database. (A,B) ROC curves of the training set and test set. (C,E) Kaplan-Meier survival curves of the two sets. (D,F) Heatmaps of the two sets used to compare the differences between high- and low-risk groups.
